# The ethical and legal landscape of brain data governance

**DOI:** 10.1371/journal.pone.0273473

**Published:** 2022-12-29

**Authors:** Paschal Ochang, Bernd Carsten Stahl, Damian Eke

**Affiliations:** Centre for Computing and Social Responsibility, De Montfort University, Leicester, United Kingdom; University of Oregon, UNITED STATES

## Abstract

Neuroscience research is producing big brain data which informs both advancements in neuroscience research and drives the development of advanced datasets to provide advanced medical solutions. These brain data are produced under different jurisdictions in different formats and are governed under different regulations. The governance of data has become essential and critical resulting in the development of various governance structures to ensure that the quality, availability, findability, accessibility, usability, and utility of data is maintained. Furthermore, data governance is influenced by various ethical and legal principles. However, it is still not clear what ethical and legal principles should be used as a standard or baseline when managing brain data due to varying practices and evolving concepts. Therefore, this study asks *what ethical and legal principles shape the current brain data governance landscape*? A systematic scoping review and thematic analysis of articles focused on biomedical, neuro and brain data governance was carried out to identify the ethical and legal principles which shape the current brain data governance landscape. The results revealed that there is currently a large variation of how the principles are presented and discussions around the terms are very multidimensional. Some of the principles are still at their infancy and are barely visible. A range of principles emerged during the thematic analysis providing a potential list of principles which can provide a more comprehensive framework for brain data governance and a conceptual expansion of neuroethics.

## Introduction

Neuroscience research is producing brain data [1, 2] which can be described as data generated from the direct monitoring of the brain itself [3] which informs both advancements in neuroscience research and drives the development of advanced datasets [4] in order to provide advanced medical, technological, and engineering solutions [5] such as the development of brain-computer interfaces (BCI) [5] for consumer electronics and neurowearables. These brain data are produced under different jurisdictions in different formats and as such are governed differently. Various brain data collaborative platforms have also been developed as tools for neuroscientists to share, use, study and analyse brain data [6] because the brain is considered to be the most important and complex human organ [7]. Considering that many of these data have different levels of sensitivity [3] and are multidisciplinary in nature stemming from multiple species and organisms [8], the application of brain data across different jurisdictions for a variety of purposes present various challenges to researchers and collaborations [1, 9] especially due to the lack of a clear data governance framework for brain data which should depict ethical and legal compliance [2, 8].

Data governance itself is influenced by various ethical and legal principles which underline the moral and legal obligations when managing data [10] and has been considered as an essential catalyst in highlighting the ethical and legal issues that needs to be addressed to advance neuroscientific collaborations through ethical neuroscience and neuroethics [11–14]. However, it is still not clear what ethical and legal principles should be used as a standard or baseline when managing brain data [14, 15] due to varying practices and evolving principles. Therefore, this study asks *what ethical and legal principles shape the current brain data governance landscape*?

Our analysis of 89 articles reveals 24 overarching principles in the brain data governance landscape which include Consent, Privacy, Trust, Transparency, Fairness, Protection and Security, Engagement, Ownership, Accountability, Autonomy, Integrity, Confidentiality, Anti-Discrimination, Beneficence and Non-Maleficence, Dignity and Respect for Persons, Legal basis, Bias, Justice, Solidarity, Responsibility, Proportionality, Independence, Retention and Destruction, and Neurorights.

This study provides a key contribution to neuroscience research and innovation by providing additional insights into the foundational principles that can shape the practice and implementation of data governance in the context of brain data. The study will inform researchers and research institutions, brain research initiatives and projects, governmental and intergovernmental organizations, funding organisations and other relevant stakeholders involved in the advancement of brain data and neuroscience research. With the application of brain data in the development of neurotechnologies such as direct to consumer neuro-wearables gaining momentum, this study also brings important ethical and legal discussions under the radar of industry leaders at the forefront of brain data related technologies.

This paper structure is as follows; the next section presents an overview of the concepts which forms the foundation of this study. This includes a discussion around brain data, its complex nature and application and how they raise multiple ethical challenges. This is followed by an overview of data governance and principles of data governance in the context of brain data and neuroethics which is followed by a methods section. The methods section also covers the literature search strategy and analysis. This is followed by the results of the analysis, discussion of results and conclusion.

## Concepts

To provide a background of the study an overview of brain data, data governance, and neuroethics is presented in the next section to frame an initial understanding of the overarching concepts that support the focus of the study.

### Brain data

The field of neuroscience has witnessed the advancement of various brain related projects and the development of brain data platforms for the purpose of collection, storage and analysis of brain data [4]. Some of these brain data have been used for research in various initiatives [2] to study the complexity of the brain and to advance biomedical research. There are many forms of data based on the reflection of processes in the human brain therefore making the definition of brain data complicated. However, in a bid to simplify brain data it can be described as data coming from direct monitoring of the brain itself [3]. Brain data can be complex and can be distinguished from other biomedical data because it is multidisciplinary in nature bringing together researchers from psychology, anatomy, medicine, and computational science. By bringing together these multiple disciplines different types of brain data are generated with different techniques and modalities thereby increasing complexity [8]. As researchers use advanced techniques in the generation of brain data, state of the art technological infrastructures are also required for the storage and processing of brain data. Researchers are no longer dealing with gigabytes or terabytes but with petabytes of brain data with a 20 minutes neural activity capable of generating about 500 petabytes of brain data [1]. Curating, storing, and moving such large complex data becomes challenging and this sometimes requires multiple storage infrastructures which also needs to circumvent cross national boundaries and jurisdictions if sharing is to take place. Due to this complexity of brain data, there is a perception that current data protection regulations and governance frameworks which apply to normal data cannot be applied to brain data because it exhibits complex characteristics. With the advent of advanced neurotechnological devices as well there can be uncontrolled and commodification of brain data thereby creating social, ethical and legal challenges due to the collection, storage and analysis of an individual’s brain data using consumer-directed neurotechnological devices [4]. Research aimed at driving the responsible use of big brain data has pointed out that there has been an inadequate classification of different brain data types and their sources especially biomedical data with respect to a proportional regulatory framework [3]. The presence of a classification especially with regards to sensitivity will promote better privacy and access strategies. In the absence of this inadequate classification, identifying and understanding the principles associated with brain data will lead to better governance of brain data.

### Data governance

In the last two decades, data governance has gained popularity because data serves as a critical element in the operation of organisations [10]. Data influences both strategic operations in any organisation and enhances decision making and collaboration [16]. The governance of data has become essential and crucial resulting in the development of various governance structures to ensure that quality of data is maintained. Data governance has had multiple definitions and each description of data governance varies greatly with the related discipline [2] with no clear universal definition. An analysis of various definitions of data governance shows an alignment with business organisations rather than research environments [17, 18]. Otto [18] defined data governance as a framework in a company in order to assign tasks and decision related duties for the purpose of handling data adequately as a company asset, while Khatri and Brown [19] defined data governance as who holds the decision rights and is held accountable for an organization’s decision-making about its data assets. Data governance definitions have also been influenced by information and communications technology and information system governance [20]. Weber *et al*. [17] pointed out the lack of academic definitions of data governance and adopted an IT governance definition which defined data governance as the framework that specifies decision rights and accountabilities to encourage desirable behaviours in the use of data. This definition is also reflected by Weill and Ross [21] who derived the definition out of the context of corporate governance. In all definitions there is an acknowledgement that data is an asset. However, the field of neuroscience generates brain data which reflect unique properties and is inconsistent with regular data types in traditional information systems environments [3]. It is also possible that practices in data governance may seek to align with the adopted definition of data governance by an organisation and with organisations or researchers having different definitions, practices may differ [10, 20]. Therefore, the study aims to develop a definition of data governance in the context of brain data through the analysis of existing definitions in neuroscience which is presented in the discussion section of the paper.

### Neuroethics in the context of data governance

Data governance is influenced by various ethical and legal principles [10] which have been adopted in brain data research and discourse. Some principles underline the importance of moral rules and obligations and the importance of identifying general considerations [22] when managing data from collection to deletion. Ethics which is an important element in data management has to do with values, therefore ethical principles are considered as the basic form of guidelines in the management of data [11]. Despite an agreement that brain data research should be ethical, there has been a debate about what ethical requirements should be the acceptable standard which will depict ethical compliance [2] especially in the context of data governance. Societies have different ethical vocabularies, expectations and understanding [23], therefore principles like privacy, consent, and justice may mean different things and exist in different forms in different jurisdictions [24]. Therefore, identifying ethical and legal principles in the context of brain data governance is essential and justifies the definition and importance of neuroethics as not just merely a division of bioethics but as a distinctive field with concepts peculiar to neuroscience research.

The broad definition of neuroethics which is concerned with the ethical, legal and social policy implications of neuroscience [11] aligns with data governance and provides a theoretical perspective to the aims of this study. This is because neuroethics attempts to provide collective answers to ethical, legal and social implications of neuroscientific research [25]. Neuroethics overlaps with traditional biomedical ethics to a substantive level and all too often it is taken to be a subfield of applied ethics [26]. However, previous research has shown that neuroethical research is larger in scope and in methodological approaches and calls for a conceptual expansion of neuroethics to accommodate progress being made in neuroscience. This study aims to follow a conceptual neuroethical perspective which although may be prescriptive but is considered as primarily theoretical and foundational [26]. A conceptual neuroethical approach provides a framework for integrating both scientific, philosophical, and social concepts thereby expanding neuroethics in the context of data governance. Building on a conceptual approach will focus on the construction of scientific, ethical, social, and legal analysis and the legitimacy of diverse interpretations of concepts or principles therefore overcoming the fundamental conceptual challenges generated by the advancement of brain research. Therefore, in attempting to answer the research question *what ethical and legal principles shape the current brain data governance landscape*, the study directly aims to identify the principles that exist in the current landscape. The study also indirectly attempts to answer the call for an expansion of neuroethics but in this case in the context of brain data governance. Finally, the study provides a platform for collaborating with neuroscience to get a shared definition of key notions which are relevant to the data governance discourse.

## Methods

To answer the research question, it is necessary to understand and synthesize the nature of research evidence while identifying key concepts in the field of brain data governance that will guide future research. The focus of the study is to embody the state of the field and provide clarity on what is known about the research question and field of interest. The approach sits well with a literature review which attempts to answer questions regarding the nature of the evidence for an intervention or what is known about a concept [27] while providing a synthesized summary of all evidence within a particular domain [28]. A literature review is expected to provide insights to answer questions such as what are the key theories, concepts, and ideas in the context of the domain and how have approaches to these questions increased our understanding and knowledge [29]. To explore the research question, a scoping review [27, 28] is conducted to examine the range, variety and nature of existing evidence and ongoing research activities in the field of interest which in this context is brain data governance.

### Search strategy

This scoping review focuses on two popular databases which include Scopus and Pubmed for the collection of academic literature. Scopus is considered as the largest citation database for peer reviewed academic literature and covers a majority of information system journals where the field of data governance is usually situated while PubMed is considered as the largest database for medical and biological science literature containing about 27 million articles and accessed by approximately 2.5 million users daily [30]. This scoping review focuses on brain data which is a form of biomedical data [31] therefore PubMed provides an index of a variety of research themes in the biomedical field which enables the capture of literature containing practices in neuroscientific research. The following inclusive and exclusive criteria is developed based on the indicative search strategy.

Inclusion criteria:

- PubMed and Scopus
- Centred on data governance principles but solely focused on biomedical data, brain data or neuro data.

Exclusion criteria

- Not substantially about principles of data governance, i.e. data governance is not the topic of investigation, but referenced and relevant empirical research involving data governance but focused on other topics not related to brain data, neuro data or biomedical data
- Centred on data structure and ontologies
- Not in English Language
- Centred on governance (e.g., government policy) involving data

This study adopts the Preferred Reporting Items for Systematic reviews and Meta-Analyses (PRISMA) 2020 framework [32] which is a reporting guideline or framework designed to address poor reporting of reviews which are systematic in nature to promote reproducibility.

**Fig 1:**
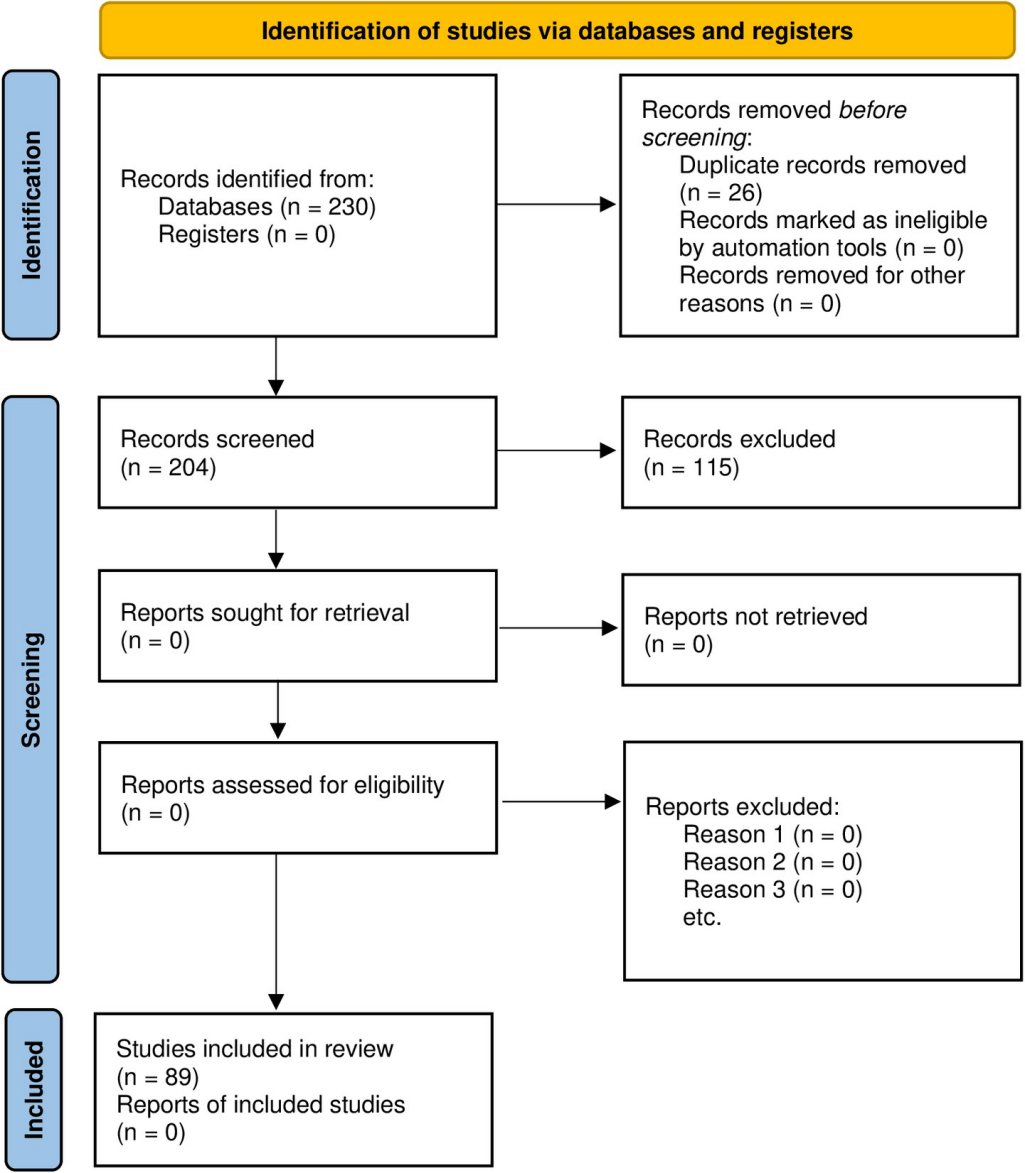
PRISMA flowchart for the identification, screening, and inclusion of studies.

The search for academic literature was carried out using the search function of the selected databases using the keyword criteria of having either “biomedical data governance or “neuro data governance” or “brain data governance” in either the abstract or title and this resulted in 230 publications. Date restrictions were not enforced in the search. The results of the search were exported into the Zotero reference management software for initial screening. The abstracts of the publications in the results were manually screened by reading to identify abstracts that fall into the exclusive or exclusive criteria. Include and exclude tags were developed in Zotero which were assigned to included or excluded publications respectively. The screening of the abstracts resulted in the exclusion of 115 publications, and after the removal of 26 duplicates the publications in the inclusive criteria were 89. Fig 1 below shows the PRISMA flowchart for the identification, screening, and inclusion of studies to be used for the analysis.

### Analysis

The 89 publications were exported to Nvivo 12 for Windows [33] to carry out a content analysis of the publications. One cycle of manual coding and one cycle of code mapping was carried out within the Nvivo qualitative data analysis software. Before the coding cycle A top-level coding scheme was developed to deductively and inductively capture the themes pre-empted by the focus of this study as follows.

*Top level coding scheme*

- Definition of Data Governance
- Ethical and Legal Principles
- Type of Biomedical Data
  ◦ Neuro/Brain
  ◦ General

The study intended to capture definitions of data governance by identified literature to provide insights on the different definitions of data governance in the context of brain data or neuroscience.

For the theming [34] of ethical and legal principles two iterations of theming were carried out and we relied deductively and inductively on normative ethical literature. These includes baseline ethical principles used in data ethics, data governance, and biomedical ethics. Principles used in the field of neuroscience ethics stem from normative ethical concepts [11, 35]. These principles are reflected in the complex mechanics of various international declarations, policies, and regulations [36]. This study takes the view that none of these principles provide a normative direction in isolation, therefore they must be interpreted with reference to how they interact with one another and other principles which are not normative but are considered to be essential in the management and governance of data. During the coding cycle one of the authors intensively mapped and tagged relevant text inductively to the themes while also inductively identifying new themes. The final themes were then inspected by two researchers with primary expertise in ethics to validate the single coding cycle.

The type of biomedical data coding process was used to highlight the articles and the respective biomedical data fields which they addressed. This approach was used to capture how much research carried out in data governance focused on brain data or neuro data.

## Results

Following the content analysis of the included publications using the coding scheme designed above, the results of the analysis are presented below.

### Definition of data governance

Out of the 89 articles analysed only four [A 2, 14, 37, 38] provided a definition of data governance. This highlights the contribution of this study and shows that there is need for more contribution to the definition of data governance in the context of brain data. Holmes *et al*. [A 37] defined data governance as the high level, corporate, or enterprise policies or strategies that define the purpose for collecting data and intended use of data. They went further to specifically define data governance as the process by which responsibilities of stewardship are conceptualized and carried out, where such stewardship may include methods for acquiring, storing, aggregating, de-identifying, and releasing data for use. Stahl *et al*. [A 14] defined data governance as all processes related to the collection, storage, processing, curation, use, and deletion of data. They also went further to state that data governance refers to who holds the decision rights and is held accountable for an organization’s decision-making about its data assets. Fothergill *et al*. [A 2] defined data governance as a strategy for the overall management of the availability, usability, integrity, quality, and security of data to ensure that the potential of the data is maximised whilst regulatory and ethical compliance is achieved within a specific organisational context. Willison *et al*. [A 38] defined data governance as the overarching polices and processes to optimize and leverage information while keeping it secure and meeting legal and privacy obligations, in alignment with stated organizational business objectives.

### Type of biomedical data

Out of the 89 articles analysed only 11 explicitly focused on brain data based on their title. This shows that in the context of data governance, further contribution is required from the field of brain data. This also justifies the important contribution of this study due to the fact it contributes to the understanding of data governance in the context of brain data.

### Ethical and legal principles

A total of twenty-four overarching ethical and legal principles emerged from the analysis of the publications as illustrated in Table 1 below.

**Table 1:** Ethical and legal principles identified.

PRINCIPLE	NUMBER OF DOCUMENTS
Accountability	11
Anti-Discrimination	7
Autonomy	10
Beneficence and Non-Maleficence	6
Bias	5
Confidentiality	8
Consent	45
Dignity and Respect for Persons	6
Fairness	12
Integrity	9
Justice	5
Privacy	26
Proportionality	3
Protection and Security	12
Retention and Destruction	1
Transparency	14
Trust	21
Neurorights	1
Solidarity	5
Independence	3
Responsibility	4
Engagement	12
Ownership	12
Legal basis	6

While the study notices that no single principle exist across the entire publications analysed, it could be noticed during the thematic analysis that some of the principles are reflected in the publications in various dimensions. This also include variations in the conceptualisation and recommendations reflected in the publications. Therefore, we present a thematic evaluation below to illustrate the findings under each identified principle.

#### Accountability

Accountability [A 38–48] according to most of the sources refers to mechanisms through which an organization makes itself answerable for its operations which includes being capable of giving account to stakeholders for the actions it has undertaken [A 46]. Some sources also point out that it also involves answering stakeholders when they ask for explanations about the conduct of the organisation or research and be under the condition of being affected by stakeholders’ judgment of the operations of the organisation. Accountability also demands that an organisation or a research body clearly identifies and discloses who is accountable for the management of data [A 43]. Some of the sources present accountability in terms of data use [A 44–46, 48] while some highlight accountability in terms of organisational accountability [A 43, 46]. Some sources also reference how the principle is reflected in data laws, for example, the GDPR’s principle of accountability of data controllers moves the onus of proof onto data controllers, thereby reducing the need for data subjects to demonstrate causation in many contexts [A 47]. Sources state that robust and transparent accountability mechanisms provide ways to ensure the precise identification of responsibility for data uses and their consequences on research subjects and key actors in research. Furthermore, accountability provides mechanisms for communicating health relevant information to data subjects [A 45]. Sources also agree that accountability can increase public trust in situations such as data reuse [A 48, 49]. One author pointed out that it is necessary as a good governance practice to establish an accountable decision-making body that can provide assurances that data are being used and linked appropriately and responsibly [A 44]. However, some of the sources stated that some biomedical institutions such as biobanks do not offer clear indications as to how the organisation renders account of its operations to interested stakeholders and the public in general as part of their governance practices [A 46].

#### Anti-discrimination

References to anti-discrimination [A 42, 47, 50–54] focuses on anti-discrimination along with equitable treatment as central principles of international human rights law. Sources also focus on profiling and racial discrimination which may be caused by data processing. Human rights instruments must allow for meaningful sanctions for persons and organisations who misuse personal data [A 51]. In the European context for example, the GDPR provides protection against the processing of data which may cause discriminatory effects on people on the basis of racial or ethnic origin, political opinion, religion or beliefs, trade union membership or health status [A 47] and categorises health data as special data [A 55]. Some sources suggest that with the advent of machine learning algorithms it is still possible that not all potential discriminatory effects will be reasonably foreseeable to allow pre-emptive action due to the way such algorithm works [A 47]. Sources also state that it is also possible that these non-foreseeable discriminatory effects are based on cultural assumptions and it is known that cultural assumptions are sometimes considered to be statistically invalid and somewhat based on ideology and can therefore be discriminating [A 54]. Furthermore reference to anti-discrimination shows that laws have often been considered to be ineffective due to the fact that it can only be applied when it can be proved that a decision was made on discriminatory presumptions which is usually difficult [A 54].

#### Autonomy

While there was no clear definition of autonomy [A 36, 43, 53, 56–62] provided, most of the sources pointed out situations that could undermine autonomy. For example one author pointed out that the use of patient data without transparency or consent may be seen to violate the principle of respect for autonomy [A 60] while the other states that decisions about data that describe who we are and how we live do indeed concern us directly and not being able to make those decisions can thus undermine our autonomy [A 43]. References to autonomy by some of the sources pointed out that there has been a focus on the use of privacy as a principle for promoting autonomy [A 58, 61]. One of the sources pointed out that research on guidelines on data sharing issued over the past two decades identified autonomy and privacy of people, and the quality and management of their data as the three most common themes [A 61]. This is supported by another author who stated that anonymisation often is portrayed as an effective mechanism to guarantee both privacy and autonomy [A 43]. While another argues that they are two core principles in the law and ethics of biomedical research which include informed consent and ethical approval and both are aimed at safeguarding the right to autonomy and privacy [A 53]. Another argues that assessing what is ethical in data access goes beyond a simple opposition between being open and protection of the autonomy and privacy of data subjects [A 57]. Laws that promote autonomy usually reference the principle of justice as pointed out by one of the authors [A 36]. One of the sources argued about the limit of neural processing pointing out that neuroscience is currently only beginning to understand how meaning is represented in the brain and it will be difficult to build machines that act autonomously [A 62].

#### Beneficence and non-maleficence

According to the sources the principle of beneficence and non-maleficence [A 36, 41, 56, 59, 60] promotes the welfare of research participants and seeks to protect participants from individual or future harm. Beneficence is usually considered as the primary goal researchers should abide by especially towards research participants who are vulnerable as deduced form the analysis. One source defines beneficence as researchers having the welfare of research participants as a primary goal, particularly those who are vulnerable[A 41]. References to beneficence state that medical ethicists seem to have certain problems with finding a moral basis for beneficence [A 56]. Sources also point out that beneficence promotes public participation and data sharing. One of the sources argues that studies have shown that there is a widespread willingness to share data for secondary purposes based on the fact that it promotes the common good and this willingness and belief are due to the fact they may be a general expectation that members of the public involved in the generation and storage of brain data can contribute to each other’s welfare by data sharing [A 60]. One author argues that the public therefore might feel justified in objecting to irresponsible, insecure or unclear future use of data which puts its members in a vulnerable state and is likely to cause individual or future harm of unknown magnitude which is a direct violation of the principle of non-maleficence [A 60]. One of the sources stated that potential participants in research usually have no say on the good being pursued in a type of research and are usually in a dilemma when considering whether or not to support a particular research based on the mission statement of the research [A 36].

#### Bias

From the analysis two major definitions or descriptions of bias [A 4, 56, 63–65] are provided. The first described bias as systematic skews in the way data is collected, annotated and categorised [A 4] while the other defines bias as any systematic error that affects the estimates of the association under study, and can emerge from the identification of subjects [A 63]. From the analysis, references to bias is usually made citing the examples of observation bias and selection bias [A 4, 63, 64]. One of the sources points out that there is sufficient evidence that observational research often leads to bias especially for less privileged groups [A 56]. Some of the sources argue that with the advent of large data repositories and data silos, data bias may be inevitable because bias operates at the level of human cognition [4] which may result to the creation of implicitly biased models and can limit data generalisation and the creation of new data [A 65]. Sources also argue that more often constructs such as race and ethnicity are being used to carry out research about a people and not by the indigenous people themselves [A 66] which results to such constructs being used uncritically when dealing with brain data.

#### Confidentiality

Preservation of confidentiality [A 39, 43, 51, 57, 59, 67–69] is viewed by the analysed sources as a cornerstone of good practice in brain banks or brain data repositories [A 68]. In order to maintain a confidential biorepository containing brain data, confidentiality and security are considered as essential [A 69]. Confidentiality, use restrictions and security procedures are applied to mitigate incidents that may undermine trust in repositories [A 70]. Confidentiality generates areas of concern in the context of data sharing which include physical security, handling of identifiers and transfer of both samples and information [A 67] and storage. Confidentiality may also generate arguments when used in conjunction with consent [A 67]. For example, deciding whether donors of data or tissue samples should get feedback where research reveals a serious medical condition is sometimes conflicting with the original intended use and consent of such data and the obligation to confidentiality. References to confidentiality by sources also highlight privacy as an accompanying principle [A 39, 51, 57, 59].Some sources argue that the obligation of confidentiality when not clearly provided by law should rests with the organisation or institution involved in the usage of data either in its policies or guidelines [A 67]. A study which was conducted to determine if Research Ethics Boards (REBs) provided ethical guidance in research involving stored biological specimens revealed that, although most of them mentioned the issue of confidentiality, fewer than 30% suggested steps for protecting confidentiality [A 67].

#### Consent

According to most sources Consent [A 4, 14, 15, 36, 39, 41–47, 50–54, 56–60, 64, 65, 67, 71–90] to having a participant’s biomedical data used in research forms a basis for best practices and codes of conduct. Consent serves as the cornerstone of bioethics and is constantly reflected as a core principle in law and ethics of biomedical research promoting autonomy [A 36]. Consent involves obtaining valid, freely given, informed, specific, unambiguous approval from a data subject indicating the data subject’s agreement to the processing of his or her personal data [A 80]. One of the sources argues that when it comes to research involving humans, consent has arguably been the dominant ethical doctrine with concerns being expressed about the consent obsession [A 88]. Our analysis identified the following major consent models which include

- Specific or Traditional consent [A 36, 65, 80, 84]
- General or Blanket or Broad [A 36, 43–46, 58, 64, 74, 80, 89],
- Dynamic consent [A 15, 36, 43, 75, 82], and
- Altruistic consent [A 76, 90].

Some sources also argue that when it comes to brain data it is possible current consent models may not sufficiently protect privacy [A 71]. It is possible for data subjects to misunderstand the full purpose of a brain scan and may sometimes confuse it for being for therapeutic purposes therefore giving rise to the problem of therapeutic misconception [A 71]. The above consent models also present challenges in various scenarios for example traditional or specific consent is challenged by future oriented research infrastructures because it is not possible to disclose to the data subject the entire range of researchers and research projects that will make use of a participants data [A 15]. General, broad or blanket consent involves consenting to a general governance framework rather than a specific research purpose [A 80]. It is also consent given to an unspecified range of future research subject to minor process restrictions [A 46]. While this promotes data reuse [A 91, 92] for secondary purposes it also leaves room for misuse and has a problematic relationship with the principle of autonomy because it is unclear what is being consented at the initial stage of the consent process. Dynamic consent involves recontacting and maintaining a steady evolving communication with participants preferences which is tailored to promote individual control. Although this is an adaptive process [A 43] it is heavy reliant on resources and may not favour data use for secondary purposes [A 15]. Altruistic consent involves consent by data subjects to process personal data relating to them, or authorisations of other data custodians to allow the use of their non-personal data without seeking a reward, for purposes of general interest, such as scientific research purposes or improving public services [A 90].

An important debate about consent also centres on the opt-in and opt-out models [A 4, 43, 60, 71, 76, 81, 83] being adopted. Extensive opting-out options in research may result in the bias of datasets and may influence the reproducibility and reliability of results [A 43]. An opt-in model is seen as a more ethical approach to promoting autonomy according to one source [A 81] but is not usually considered as a more scientifically valid approach for population based studies. Sources argue that with consent moving from paper-based form to a more electronic ecosystem [A 43, 86] many services have a default opt-in policy with regards to the use of personal data rather than an opt-out policy.

#### Dignity and respect for persons

While there was no definition of dignity and respect for persons [A 41, 50, 52, 53, 67] most of the sources mention words like autonomy, wellbeing and safety in association with dignity and respect [A 41, 52, 53]. With some saying that it entails that research participants should be valued and respected and they should be respected both as beings who are capable of exercising decisions, and also as members in communities who make choices in the context of their relationships [A 41]. Some argue that in Europe the GDPR seeks to secure the dignity of people in terms of specific rights [A 61] which will in turn promote autonomy and privacy.

#### Fairness

The analysis provided the principle of fairness in two dimensions which include fair access and procedural fairness [A 14, 39, 41, 42, 48, 51, 52, 76, 84, 93–95]. Fairness in terms of fair access has to do with the requirements for users being allowed to look at and use the data [A 14], and should address concerns about fair or equitable access to data, research results, and public health benefits derived from those results [A 51]. Some authors state that fairness may involve using the FAIR principles [A 96] to achieve FAIRification of data [A 95]. This sometimes means that Individuals should be provided with a simple and timely means to access and obtain their individually identifiable health information in a reliable form and format [A 42]. Governance frameworks may allow for information to be exchanged in certain scenarios including data subjects or research participants being able to access their own biomedical data e.g via patient portals to facilitate fair access [A 84]. However, how custodians or data stewards or even clinicians react to such access by patients and other data consumers need to be addressed in governance frameworks to promote fair access. According to one source the use of data access committees may be a good strategy to promote oversight of fairness in data access [A 44]. However one author highlighted that one of the most time-consuming tasks in the access process is obtaining the necessary local scientific and ethical approvals [A 97]. According to the sources Procedural fairness [A 41] involves fairness in processes related to regulatory treatment of research projects [A 51].

#### Integrity

The analysis presented integrity [A 38, 41, 42, 52, 53, 62, 67, 75, 94] to be two dimensional with the first being towards protecting the wellbeing of research participants and the second involving maintaining data quality. While there is a need to request for additional samples from the same participants and the storage of these additional samples in certain repositories for an indefinite number of years, there is thus an increasing need to deploy adequate measures of confidentiality and protection of the integrity of the research participants without jeopardizing information that could benefit the entire research process [A 67]. Some of the sources states that to achieve data integrity, data must also be findable, accessible, interoperable and reusable thereby following the FAIR principles of data management [A 95, 98]. If samples are used in a research project, they must be collected, stored, and processed in a way that preserves their long-term stability, searchability, and integrity in order to reduce data waste [A 41]. Some of the sources state that standardisation of high quality data brain data [A 99] and how data should look like should be enforced at a curatorial level which will serve as a measure for building community standards and maintain integrity [A 94]. Persons and entities should take reasonable steps to ensure that research information is complete, accurate, and up-to-date to the extent necessary for the person’s or entity’s intended purposes and has not been altered or destroyed in an unauthorized manner [A 42]. In order to promote integrity, Individuals or entities should be provided with a timely means to dispute the accuracy or integrity of their individually identifiable information, and to have erroneous information corrected or to have a dispute documented if their requests are denied [A 42]. One author points out that appropriate measures need to be taken to maintain the integrity of brain research due to situations like dual-use of neurorobotics for civilian and military applications [A 62].

#### Justice

Justice [A 4, 36, 41, 60, 76] according to one of the sources can be defined in terms of the widely held and acceptable legal safeguards which ensure that decision makers conform to a pattern of procedural fairness that ensures that the rights of research participants or stakeholders are respected so that public confidence in the decision making process is maintained [A 36]. This definition is referred as natural justice by the authors. Refences to justice also point out that the principle of justice ensures that the benefits and burdens of a research project should be distributed equitably among all groups in society [A 41]. The use of data for private gains and for commercialisation or private use as pointed out by some sources [A 49] is considered to be in violation of justice because it is unfair to use data for purposes other than the original intended use [A 60]. This concern of misuse and violation of justice continues to resurface especially when it comes to how private organisations use brain data. Sources also point out that the processing and benefits of biomedical big data may be placed on specific social, cultural and economic groups and it may be possible to express such divides as ethically problematic in terms of justice [A 76].

#### Privacy

References to privacy described how the anonymity of participants from whom data is collected are protected [A 14]. Privacy is usually used to describe the preservation of a participant’s integrity and the ability of participants to have control over what they reveal about themselves and includes concepts of appropriate protection and use of information [A 100]. Some of the sources emphasize that privacy preservation in data collection and integration is essential when handling big health data [A 98, 101] due to the fact that there is always an ethical tension between the need to share data and maintaining privacy in the process. Although the sharing of data is largely seen as being for the overall common good, it also has the potential to create new risks, and increase existing ones [A 60]. References to privacy also highlight whether existing privacy regulations (ie, the Health Insurance Portability and Accountability Act, or HIPAA) provide sufficient protection to patients while balancing access to data for researchers [A 102]. The various perceived risks involved in sharing brain data which may lead to privacy concerns may include routes to harm like neurohacking [A 4], leakage or loss, unauthorised access, errors in repository records and aggregating data to a group’s disadvantage. One of the authors stated that collection and commodification of neural data that may put vulnerable individuals at risk with respect to the privacy of their brain states can be considered as a threat to neuroprivacy [A 4]. Therefore achieving the highest level of data safety and data privacy is crucial [A 103].

Sources recommend the following approaches to privacy: privacy of personal information, privacy of the person, privacy of personal behaviour and privacy of personal communications [A 100]. This can be extended by adding the following privacy interests and concerns for biobanks and repositories: physical privacy (example may include gathering and storing biospecimens and testing them without consent), informational privacy (such as possible misuse of information), decisional privacy (e.g., control or influence over what is done with data and biospecimens), and proprietary privacy (e.g., ownership of biospecimens and the control of identity) [A 15]. Sources argue that as more data sources become available such as data from consumer neurotechnological devices and advanced analytics being applied for various purposes, protecting privacy is becoming a complicated task. What contributes to this complication and complexity is that standard mechanisms of protection such as anonymisation, de-identification, pseudonymization, notice and consent are excessively stretched in this environment of new capabilities [A 45]. Also a substantial difference in data sharing and data regulations of different countries contributes to the complexity of providing privacy [A 104]. References to privacy also highlights adopting privacy as a feature by design as a good practice [A 84, 87].

#### Proportionality

According to some of the sources proportionality [A 39, 41, 53] involves balancing the risks and benefits of research and the entire research process. According to one of the sources proportionality involves ethics review and oversight being commensurate with the risks and benefits for research participants [A 41]. Some of the sources indicate that a governance framework must contain processes that are proportionate, consistent, and targeted only at cases in which action is needed. These include it being clear, coherent, efficient, effective, proportionate, and properly targeted so that the system is neither over-inclusive nor under-inclusive, nor unduly onerous where differing levels of regulation are warranted [A 39].

#### Protection and security

From the results of the analysis, references to protection focused more on the protection of research participants than on the protection of data, while references to security focused more on the security of infrastructure and data [A 4, 36, 39, 41, 42, 45, 52, 67, 94, 105, 106]. One of the sources recommended that security should be a guiding principle in ethical frameworks and described security as state-of-the-art measures which must be employed to minimise the risk of research projects’ data becoming lost, misused, or unjustifiably altered or destroyed [A 41]. Some of the sources recommend that data should be protected with reasonable administrative, technical and physical safeguards to ensure its confidentiality, integrity, and availability and to prevent unauthorized or inappropriate access, use, or disclosure [A 42]. Sources also identify that the security and protection of data has always been a challenge, with cyber-attacks, hacking of databases, and data kidnapping being reported frequently [A 4, 45]. Some of the arguments point out that protection and security measures can be put in place to prevent such misguided uses of data, but they are often perceived by researchers as constraining users’ freedom to analyse the data as they see fit [A 105]. References to security, protection and privacy are high priorities for research participants as highlighted by some sources, particularly when it is proposed that data may be shared with commercial companies and governmental organisations [A 106] with research participants pointing out that governance and security is what will build trust and not consent and privacy which is usually talked about [A 94].

#### Retention and destruction

References to retention and destruction focused on what manner brain data is stored, how long such brain data is stored and whether such data must be destroyed after use [A 14].

#### Transparency

Some of the sources describe transparency as the availability of information about an actor that allows other actors to monitors the working performance of such actor [A 46] while some describe transparency in the context of governance as the accessibility and visibility of the governance structures of consortia [A 107]. Sources also referred to transparency as openness [A 42, 107, 108] References to transparency include using community engagement activities to promote trustworthiness and visibility [79, 106, 108] and using transparency to strengthen public confidence in institutions or research projects which ensures accountability while facilitating public trust [A 41, 46, 48, 54, 79, 108]. This can include sharing information about the proposed use of data, expected societal benefits, harm-minimisation strategies, degree of security and encryption and research results [A 16, 108]. Transparency is almost ubiquitously regarded as a positive feature of itself and is believed to be a mechanism to facilitate accountability and participatory governance [A 38, 46, 48]. Sources also emphasize that greater transparency is needed in safeguarding of data and in data sharing agreements [A 60, 107, 108]. Transparency is considered as benchmark in assessing the adequacy of good regulation for databases housing biomedical data [A 39]. Further recommendations to achieve transparency as suggested by some sources include making all policies, decisions, and practices regarding data used in a particular initiative freely accessible by members of the public in an understandable format [A 38].

#### Trust

From the analysis, references to trust focused highly on the call for trustworthiness [A 36, 45, 46, 79, 88, 94, 105] and transparency [A 36, 41, 45, 60, 88, 94]. One of the sources argues that trust underscores data governance policies and involves respect for human autonomy, prevention of harm, fairness and explainability, while using a human-centred approach to building and sustaining trust [A 61], while another points out that mechanisms to increase public trust include transparency of motivation, data handling, and data flow [A 60]. References to trust is also multi-dimensional, complex, and usually varies according to different context. Furthermore, from the analysis it was seen that there are many recommendations on how to achieve trust. Some of the sources state that common qualities of trust and trustworthy institutions include:

- Integrity (which means that the institution is fair and just)
- Dependability (this depicts that the institution will do what it says it will do) and
- Competence (the institution has the ability to do what it says it will do) [A 79]

Existing bioethical literature emphasize the importance of public trust in data production, collection, usage, and sharing [A 36]. References to trust also show that research participants views on storing and using their data is linked to the kind of trust or distrust the public has in an organisation or individual who is using and accessing their data. Participants distrust towards organisations who handle their data generally occurs along two dimensions. The first is distrust of a party’s ability, or competence, to ensure data security, while the second is distrust of a party’s motivations [A 60]. However, there is an agreement on trust with regards to using societal benefits as a justification for sharing data [A 78, 83]. To merit and garner trust, guardians of citizens’ health data ought to ensure that they respect the values of the people who are expected to trust them with their data. Sources also point out that the public or research participants should not have any fear that they are being manipulated into sharing their health data [A 78]. Lack of public trust in the use of data is important and can derail large scale initiatives and a typical example as provided by the analysed sources is the UK care.data initiative which shows how mistrust on the part of the public can derail large-scale data initiatives [A 45].

#### Neurorights

Neurorights featured in only one of the articles analysed. References to neurorights provided questions around if brain data should be treated as any other type of biometric data or should be treated as a special type of data with special rights [A 4].

#### Solidarity

While there was no definition of solidarity [A 36, 47, 56, 76, 83] provided by the sources, solidarity was mostly referenced together with justice and consent. According to one of the sources, solidarity displays a dominant emphasis on people’s willingness to engage in activities that benefit others [A 36]. One of the sources argue that new approaches to big data governance should be based on the principle of solidarity [A 47] and that they should include three main pillars to reflect solidarity which includes placing greater emphasis on whether or not specific instances of data use are in the public interest, strengthening of harm mitigation instruments, and developing new legal mechanisms to ensure that significant parts of financial profits created on the basis of data use go into the public purse [A 47].

#### Independence

While there was no clear definition of independence A [38, 44, 109] by the sources, there were recommendations of what independence should entail. One of the sources highlights independence as a guiding principle to their recommended governance process [A 38]. One of the references pointed out that one way of safeguarding the right to privacy and the right to benefit from science is to ensure a robust and independent data access process for complex and sensitive research resources as this may reduce data hoarding [A 44]. Another source recommended that to promote participatory governance all actors involved in the governance process should be able to able to operate in a zone of bounded independence and a good governance process should be able to operate independently from management to ensure that decisions are free from institutional conflicts of interest [A 38]. Another source points that independence from funders is considered as a crucial element of governance and promotes the trust and participation of the public in research initiatives [A 109]. One source recommended that through the use of Independent committees who make determinations about new uses of data, practices related to hoarding of data or data hugging by researchers may be reduced [A 44].

#### Responsibility

The idea of responsibility [A 2, 52, 62, 107] as presented by most of the sources is to provide a principled and responsible approach to research and data governance which is reflective of societal needs. A major approach in achieving responsibility as pointed out from the analysis is the concept of Responsible Research and Innovation (RRI) which is an approach to governance that aims to unite and respond to various stakeholders and their expectations [A 2]. RRI includes components which improve the dynamics of research and innovation such as public engagement, open access, gender equality, ethics, and governance [A 107]. According to one source RRI can hold governance to high ethical standards bridging the communication between researchers and society [A 62]. Sources point out that although RRI is currently gaining momentum, it lacks collective meaning globally [A 107] and is mostly an approach adopted by scientist and initiatives in the European Union on the development of technological innovations [A 62]. Sources also pointed out that with the adoption of RRI, a major challenge which involves the governance of consortia especially after the end of research projects can be overcome [A 107]. Sources agree that integrating responsibility in governance to achieve responsible data governance [A 2, 110] may structure governance in a way that promotes reflexivity, foresight and consideration of future impacts.

#### Engagement

The sources indicate that ongoing engagement [A 38, 40, 45, 59, 73, 78, 82, 85, 88, 106, 108, 111] and involvement with stakeholders, communities, researchers, and scientists is integral to the governance of data. Citizens are gradually becoming the driving forces of digital health applications, innovations and data [A 45]. The failure of the care.data initiative is a good illustration of the current boom of appeals for engagement and also shows how lack of engagement can deter large scale data intensive initiatives [A 85]. Engagement is seen as a way to foster trust especially with the public and overcome data use barriers while encouraging accountable and responsible research [A 88]. However, some sources pointed out that ways of enhancing engagement and involvement of the public and stakeholders is usually poorly articulated and defined [A 78, 85, 88]. Significant confusion exists regarding concepts such as engagement, involvement, and participation which generates confusion on the application of these concepts and makes the citizen science rhetoric to pose even a greater risk for confusion [A 78]. Furthermore, although there are some semantic overlaps between terms which involve participant-centric initiatives, the role these concepts assign to participants in research is neither consistent or clear [A 85]. Some sources recommend the inclusion of data subjects as co-decision-makers, co-researchers and as co-principal investigators stating that this can be seen as the most straightforward realisation of engagement [A 85] and may also involve the use of participant association models which may involve participant boards [A 40]. References to engagement also pointed out that in multicultural societies where the views on acceptable data use differ, research initiatives involved in the use of large data repositories may use a social licence with the parameters of the social licence changing over time as citizens became more comfortable with, or more suspicious of various uses of the research and its data [A 108]. The analysis also discovered that public discourse and engagement has been implemented as an ethical requirement in some emerging biotechnologies [A 111].

#### Ownership

References to ownership argued that the clarity of the ownership [A 14, 43, 45, 76, 81, 82, 108, 112–116] of data should be considered as an essential governance element as it provides insights to rights regarding the modification and redistribution of data, along with benefiting from intellectual property and innovations developed from its analysis [A 76]. The analysis also shows that there is a tension between ownership and access. Some of the authors stated that they has been an increase in the call for the empowerment of stakeholders (participants and researchers alike) through data ownership because this gives rights to individuals to be the guardians of their own data allowing them to access, modify and set access rules [A 82]. Another author pointed out that although there is a debate around ownership and access, recent empirical research revealed a substantial lack of availability of access policies in practice [A 115]. Moreover, with regard to access requirements, available access policies vary widely and are neither standardized nor harmonised [A 97, 99, 115]. Some of the sources argue that sometimes literature discusses ownership in terms of control [A 43, 76], while another argues that ownership can undoubtedly be viewed in terms of owning products and intellectual property, which raises future debates around the ownership of intellectual property generated form the analysis of aggregated big datasets [A 76]. Some sources also state that while the basic structure of ownership can be strengthened by a calculated emphasis on the ethical value of the protection of personality [A 113], who receives various benefits from the provision or use of data, is relevant in academically-oriented research and various ownership structures of compensation in terms of authorship, compensation, acknowledgements should be reflected in governance structures [A 14, 115]. One of the sources also suggests that policies and governance structures should reflect clear ownership elements and should not be restricted to only copyright notices [A 14].

#### Legal basis

Sources pointed out that the collection, curation, processing and deletion of data should be done on the grounds of sufficient legal basis [A 38, 53, 60, 74, 81, 90]. References to legal basis describe legal basis in governance as the foundation that ensures that appropriate legal frameworks, applicable laws, regulations, and standards are behind the reasons for certain data governance actions [A 38]. One author pointed out that some countries have been left with the choice of either requiring specific consent for research or using other legal grounds such as public interest as a lawful basis for processing data due to the tension between legal basis and broad consent [A 90]. Sources also argue that legal basis for the processing of data is important in research which makes use of secondary data because data is used for a different purpose than that originally intended for and this is important even when identifiers in the data are stripped and if individuals are still potentially re-identifiable by a pseudonymisation code [A 60]. One source also highlighted that there are key differences in the legal basis for certain actions across various jurisdictions [A 74].

## Discussion

**Fig 2:**
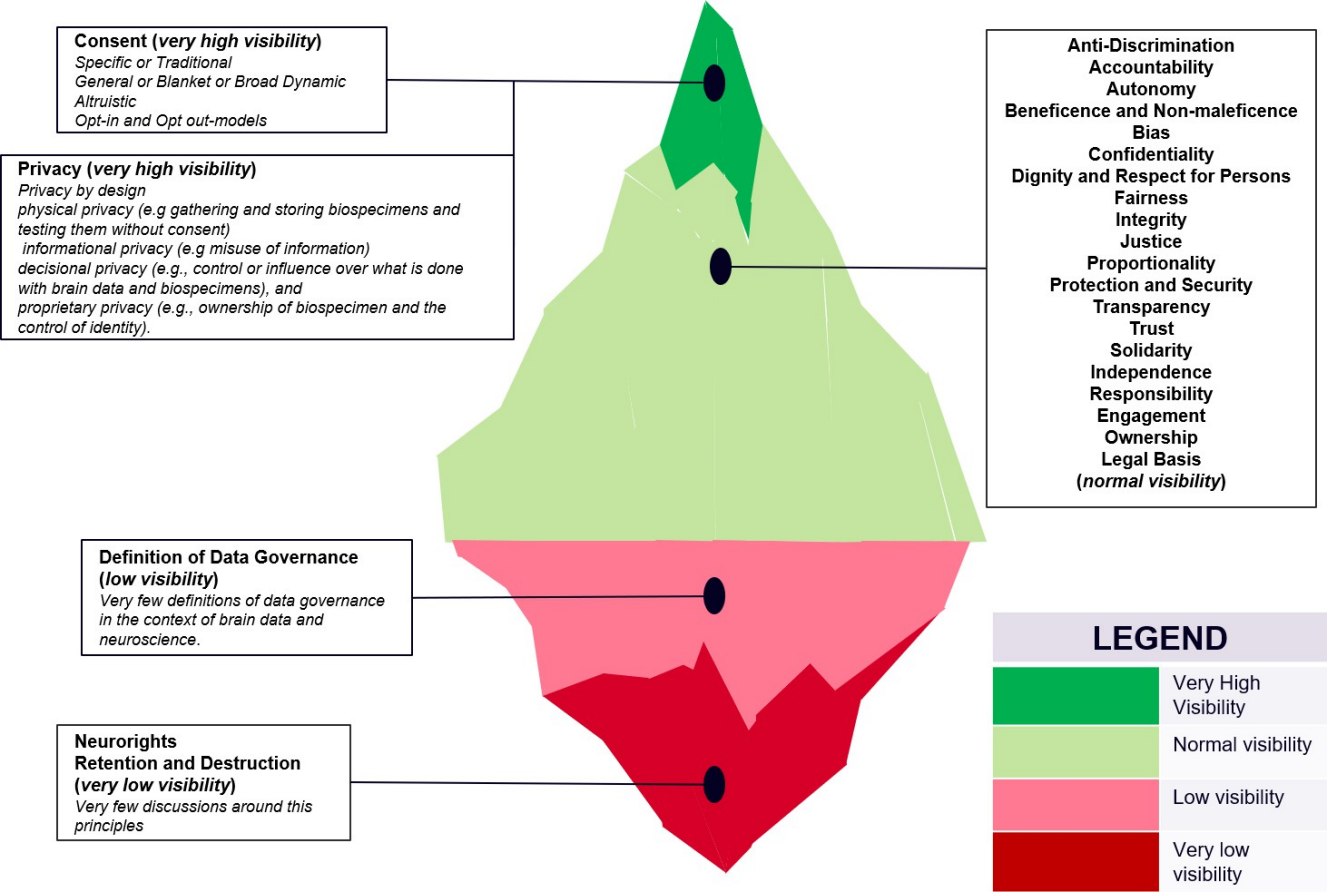
Data governance, ethical and legal principles visibility.

The results show that a lot of discussions are ongoing around most of the identified principles and are mentioned repeatedly as identified in the analysis of the study set of literature. However, the results showed that some ethical and legal principles were still at their infancy and have low visibility regarding discussions around them as shown in Fig 2 below which shows the visibility of the various principles based on the level of discussions identified during the analysis. The level of visibility was determined by the number of references to each principle and the number of sources or articles which each principle featured in. We used the top 2 most referenced principle as having the highest visibility and the least two as having very low visibility. For example, the principle of neurorights was mentioned only by one article calling for the need for special rights for neuro data. This means that the discourse around neurorights is perhaps moving at a pace slower than anticipated or perhaps neurorights is a new concept that has not been considered as an essential principle. With the advancement of neuroscience and neurotechnological devices neurorights should be an important legal discourse and it will be interesting how it is reflected in legal documents of projects associated with brain data. Questions as to whether national governments should hold exclusive rights to the processing of brain data is also another dimension to answering the questions posed by adopting neurorights. The underrepresentation of neurorights might be problematic considering recent evidence that some countries like Chile have enacted a neurorights law [117, 118]. With the emergence of data protection regulations around the world (such as GDPR, PIPEDA and HIPAA), the rights of data subjects have become more pronounced in theory and in practice [119, 120] however, there still remains a dearth of literature on neuro (data) rights. With only one reference of this principle in the reviewed literature one can deduce that either neuro researchers are slow in the uptake or the underlying argument behind the principle has not been accepted within the community. The same goes for the principle of retention and destruction of data which was mentioned rarely which raises questions about deletion policies of projects involved in the collection of brain data. The underrepresentation of retention and destruction shows that perhaps research projects, brain data repositories and stakeholders are more focused on the collection of brain data than in the definition of well laid down structures for the appropriate deletion or destruction of data at the end of consent validity. The destruction of data is important as it is part of the data management lifecycle and part of curation which is a data governance practice. Although it is challenging to provide clear retention and destruction strategies especially with the use of broad consent procedures for obtaining such data, providing retention and destruction information in policies and guidelines provides clarity on data storage strategies and procedures used for the destruction of acquired data. This is important as it highlights various practices that may be used for brain data retention and disposal. It showcases what data format is used in storing the acquired brain data, were such data is stored, how long it will be stored for, and if such data is to be destroyed after use what ethical procedure is used for destruction and disposal. This is an essential element of the brain data life cycle and may be adopted as an ethical principle for ethical governance of brain data. The underrepresentation of neurorights, retention and destruction suggest that these issues maybe flying under the radar of mainstream ethical neuroscience and data governance debates.

The results show that there are few definitions of data governance in the context of brain data. We consider this as having a low visibility as shown in Fig 2 above because the definition of data governance provides more clarity on how to advance neuroscience collaborations and practices in the management of brain data. The definition of data governance influences stakeholder activities and assists in the development of policies and guidelines. Without an acceptable definition of data governance achieving standardisation and harmonisation may be challenging for stakeholders globally when handling brain data. This is reflected in the results which shows the various identified definitions of data governance portraying different concepts. For example, a definition talks about de-identification and conceptualises stewardship as the highlight of data governance. While some highlight availability, usability, integrity as part of the governance process. By deducting the important keywords from the definitions identified, we contribute to the definition of data governance in the context of brain data by presenting a comprehensive data governance definition as follows

*Data governance can be defined as the policies and strategies that define responsibilities of accountable stewardship which include acquiring*, *aggregating*, *deidentifying*, *processing*, *curation*, *retention*, *deletion*, *use and the overall availability*, *usability*, *integrity*, *security*, *and privacy of data in alignment with ethical*, *legal*, *and social obligations*.

Our proposed definition integrates the key concepts identified in existing definitions and extends the definition of data governance for brain data. It highlights the important concepts which may be overlooked in the management of data such as the deletion of data because the future use of data is usually not clear in research projects and researchers may want to acquire data without proper data retention and destruction strategies in place. Our definition highlights the ethical, legal, and social issues that are associated with brain data and ensures that stakeholders, research consortia, ethics review boards and data stewards reflect on these issues while developing policies and guidelines. This approach embeds in data governance the need for moral reflexivity and responsibility by stakeholders involved in neuroscience research.

The study provides interesting findings in terms of the variation of how principles are presented and defined. Most of the principles are presented in multiple dimensions and interpretations in terms of how they should be applied or considered. For example, fairness is presented in some form as fair access to data by both research participants and other researchers interested in using such data. Another form of fairness is presented as procedural fairness which discusses regulatory hurdles and involves fairness in processes related to regulatory treatment of research projects. Integrity in some form is viewed as protecting the interest of research participants and in another form as only maintaining data quality. The multiple variation in interpretations justifies the call for harmonisation of concepts and standards which has been raised by various ethical commentators. The misalignment of concepts and frameworks and the inconsistent if not incomprehensible terminologies used in describing practices produces challenges in data sharing, data reuse and research practices [15, 121, 122]. The study shows that disjointed approaches to principles and practices still exist [102], and researchers must deal with these disjointed concepts which are sometimes based on local regulatory requirements. Unless there is a harmonisation of principles at an acceptable international level of concordance these concepts will continue to form challenges [123]. For example, how are researchers who access data from multiple sources which may be from various jurisdictions to know if a de-identified, coded, unlinked or pseudonymised data is equivalent to a reversibly anonymised data [15]. This issue may also affect inconsistent interpretation by research ethics committees and ethics review boards and may lead to conceptual and procedural divergencies as to which ethical and legal principles should be prioritised. Furthermore, it may provide difficulties as to how ethical conflicts may be resolved and undermine the drive for global reciprocity in scaling ethical and legal hurdles.

A particular issue of importance is the different concepts of privacy discovered in the study set of literature. Based on the results, concepts of privacy should include privacy of personal information, privacy of the person, privacy of personal behaviour and privacy of personal communications. This can be extended by adding the following privacy interests and concerns for biobanks and repositories: physical privacy (example may include gathering and storing biospecimen and testing them without consent), informational privacy (such as possible misuse of information), decisional privacy (e.g., control or influence over what is done with brain data and biospecimens), and proprietary privacy (e.g., ownership of biospecimens, datasets from brain repositories and the control of identity). Floridi [124, 125] offers a similar typology but argues that informational privacy is central. This shows that there are different perceptions of privacy which can influence privacy practices in relation to managing brain data. For example, some brain repositories may not provide user authentication mechanisms to allow access to brain data sets while some may require approval by a research ethics board and justification for use of data by the principal investigator as a form of access control. This can be challenging because it can result to different approaches to privacy. However, Privacy by design is encouraged as a good approach for enabling more opportunities as a research project progresses [84]. Privacy by design is embedded under data protection by default and by design which is enshrined under the General Data Protection Regulation (GDPR). Data protection by design involves the use of data protection impact assessments (DPIAs) and promotes the privacy of the research data and participants because the future uses of data may not be fully predicted. This ensures a proactive approach rather a reactive approach to brain data privacy and ensures that there is full protection of data in the data lifecycle. However, the GDPR is under the European jurisdiction, and it will be interesting to see how laws in other jurisdiction embed DPIAs and data protection by default in their regulations.

The results also show that there is a heavy discourse in terms of consent and there seems to be no consensus. The pro-ethical nature of consent as the cornerstone of bioethics might be the reason for its prevalent nature as consent is interwoven with the sensitive nature of brain data. The arguments about various consent models being inappropriate for various forms of biomedical and neuroscience research, especially for brain data shows that brain data should be treated as a unique type of data. Most of the arguments support the use of broad consent as an appropriate consent mechanism for research that has unknown secondary range of usage. However, results also identified misuse as a concerning factor with broad consent. To promote harmonisation and reduce the inconsistent use and interpretation of consent terms, we identified the various forms of consent based on the presentation of the analysis as, specific or traditional consent, general or blanket or broad consent, dynamic consent, and altruistic consent. Some of the research argued that the use of data for altruistic purposes should be considered as the best consent model. Another important debate about consent is based on the opt in and opt out models being used together with consent. A lack of synergy between opt in and out models may reduce the validity of consent and trust. For example, the use of electronic form of consent was highlighted as usually providing ways of opting in by default but fewer ways of opting out by default and sometimes opt out models of consent are mistaken to be ethically equivalent to informed consent and may sometimes take advantage of vulnerable persons [76]. With the development of more advanced neurotechnologies and BCI for multiple purposes, consent may become more complex especially when it is done electronically without the known future purposes of acquired brain data. Furthermore, some of the services use End User Licence Agreements (EULAs) which are difficult to understand and the services for data sharing may prove to be unusable when opting out especially when sharing brain data [4].

This study shows that there is a need to clarify the ownership of brain data at various stages of the research process. Researchers, participants, and research projects may misunderstand or misinterpret ownership due to lack of clear ownership structures which may result to lack of trust or data hoarding. It is also necessary to see how the ownership of brain data in repositories is not just limited to copyright licences or creative common licenses. This may result in making the datasets open source without due acknowledgement to the contributors of such data. To provide clarity around the arguments, considerations about ownership should include how and which data are made available, who is the owner of the data? does a participant still have governance of this own datasets? or does the researcher govern it, and if so, which researcher, or principal investigator, the data generator, or is it the person who tries to make sense out of the data? Furthermore a clear distinction between ownership and stewardship should be embedded in governance structures or policies as sometimes stakeholders do not understand the difference [112]. In terms of public private partnerships, the clarity of ownership is essential. A major concern about public private partnerships is that private partners are provided with the chance to appropriate public datasets which may involve extraction of undue value from their access to public data [108]. This raises questions of ownership of raw data and ownership of research outputs which needs to be addressed in such partnerships.

These findings have implications for guidelines and policies on neuroscience research and collaborations. With the advent of large-scale neuroscience projects such as the International Brain Initiative, there is a need for the various stakeholders in neuroscience to mutually align various brain data research agendas. This mutual alignment should be done around harmonised ethical and legal principles in order to attain ethical and legal reciprocity. A mutual alignment of principles will provide a global convergence of neuroscience practices around the identified legal and ethical principles. However, this does not imply that that to achieve global consensus the moral and cultural pluralism which identifies various practices should be abolished but should be used as an avenue for positive engagements and deliberations with stakeholders in order to understand various ethical and legal standpoints and how they should be reciprocity. Furthermore, with the diverse use of data and neurotechnological advancements such as neurowearables, brain data may not be used only by researchers or scientist who are bound to a sense of moral responsibility but by private sector initiatives and commercial interests who may use brain data for other purposes. It is therefore necessary to achieve standardisation and harmonisation using a top-down approach through the use of intergovernmental organisations, stakeholders, influential international standardisation bodies and the International Brain Initiative. Such efforts will reduce the time lag between technological innovation and ethical appropriation under the radar of equivalent regulation.

Although this paper cannot say the principles identified are exhaustive in nature; however, it provides insights on the current landscape of principles around brain data, neuroscience and neuroethics which also applies to the governance of brain data. It also complements the call for the identification, harmonisation and standardisation of principles and expands the data governance discourse by attempting to collate the principles and definitions in the current brain data governance landscape.

## Conclusion

The development of a data governance framework for the governance of brain data is a key concern in enhancing collaboration and data sharing in brain research. The premise of this paper was to identify to a large scale the ethical and legal principles used in governance discussions around brain data. By focusing on published literature through a scoping review and content analysis, this paper provides more insights on the principles that reflect governance with regards to brain data. It also indirectly attempts to carry out a conceptual expansion of neuroethics and, identification and harmonisation of principles. The identification of these principles provides insights into using these principles to design practical decision-making approaches in neuroscience which can be called an ethical principalism.

The study has several limitations. First, only Pubmed and Scopus were the databases used for the search of literature. Expanding the search other excluded databases may provide more literature to include in the analysis. However, we believe that most of the literature used have been indexed in other excluded databases due to random checks. The quality of the coding process may be a bit skewed, and it is possible some principles were unintentionally not identified due to the fact that the content analysis and coding process was carried out by one author. Our study also presents the typical limitations with regards to theory and practice when using academic literature as opposed to grey literature such as policies and guidelines. Using grey literature may provide what is in practice by projects involved in brain research. Finally, a language bias may have tilted the results towards English results and non-English publications containing information relevant to the study may have been left out

Despite the limitations highlighted, this paper provides a significant contribution through its demonstration of data governance principles in the context of brain data and an attempt to synchronise neuroethics in the context of data governance. The study discovers the visibility of data governance definitions and principles in the landscape. Finally, the study identifies arguments, suggestions, and dimensions of ethical and legal principles. Further research is required to understand how brain research projects, initiatives and consortia display the identified principles of this paper in their governance structures and policies globally. This will provide an understanding of brain data governance around the convergence or divergence of the principles identified in this paper. Determining convergence or divergence will provide clarity on the development of a brain data governance framework. Also, further research is needed to understand the key differences in private and public sector use of big brain data as this will identify how principles are applied in both sectors. Furthermore, by understanding the perceptions of stakeholders from different jurisdictions around the identified principles in this paper an understanding of brain data governance from a global cultural context can be achieved.

## Supporting information

S1 Checklist Preferred Reporting Items for Systematic reviews and Meta-Analyses extension for Scoping Reviews (PRISMA-ScR) checklist. (DOCX)

S1 File Reference list of study set used for analysis. (DOCX)

## References

[1] Landhuis E. Neuroscience: Big brain, big data. Nature. 2017 Jan;541(7638):559–61. doi: 10.1038/541559a 28128250

[2] Fothergill BT, Knight W, Stahl BC, Ulnicane I. Responsible Data Governance of Neuroscience Big Data. Front Neuroinform. 2019;13:28. doi: 10.3389/fninf.2019.00028 31110477 PMC6499198

[3] Hallinan D, Schütz P, Friedewald M, Hert P de. Neurodata and neuroprivacy: Data protection outdated? 2014;(Journal Article). Available from: http://publica.fraunhofer.de/documents/N-283673.html

[4] Kellmeyer P. Big Brain Data: On the Responsible Use of Brain Data from Clinical and Consumer-Directed Neurotechnological Devices [Internet]. 2018. Available from: https://www.scopus.com/inward/record.uri?eid=2-s2.0-85047113952&doi=10.1007%2fs12152-018-9371-x&partnerID=40&md5=60e569f21f0301e42a6d94e76fe75295

[5] Bablani A, Edla DR, Tripathi D, Cheruku R. Survey on Brain-Computer Interface: An Emerging Computational Intelligence Paradigm. ACM Comput Surv. 2019 Feb 13;52(1):20:1–20:32.

[6] Teeters JL, Godfrey K, Young R, Dang C, Friedsam C, Wark B, et al. Neurodata Without Borders: Creating a Common Data Format for Neurophysiology. Neuron (Cambridge, Mass). 2015;88(4):629–34. doi: 10.1016/j.neuron.2015.10.025 26590340

[7] Tang Y, Chen D, Li X. Dimensionality Reduction Methods for Brain Imaging Data Analysis. ACM Comput Surv. 2021 May 3;54(4):87:1–87:36.

[8] Eke DO, Bernard A, Bjaalie JG, Chavarriaga R, Hanakawa T, Hannan AJ, et al. International data governance for neuroscience. Neuron [Internet]. 2021 Dec 15 [cited 2021 Dec 24];0(0). Available from: https://www.cell.com/neuron/abstract/S0896-6273(21)00955-7 doi: 10.1016/j.neuron.2021.11.017 34914921 PMC8857067

[9] Minielly N, Hrincu V, Illes J. Privacy Challenges to the Democratization of Brain Data. iScience. 2020;23(6):101134. doi: 10.1016/j.isci.2020.101134 32438287 PMC7235278

[10] Abraham R, Schneider J, Brocke J vom. Data governance: A conceptual framework, structured review, and research agenda. International journal of information management. 2019 Dec;49:424–38.

[11] Illes J, Bird SJ. Neuroethics: a modern context for ethics in neuroscience. Trends in neurosciences (Regular ed). 2006;29(9):511–7. doi: 10.1016/j.tins.2006.07.002 16859760 PMC1656950

[12] Rommelfanger KS, Jeong SJ, Ema A, Fukushi T, Kasai K, Ramos KM, et al. Neuroethics Questions to Guide Ethical Research in the International Brain Initiatives. Neuron (Cambridge, Mass). 2018;100(1):19–36. doi: 10.1016/j.neuron.2018.09.021 30308169

[13] Adams A, Albin S, Amunts K, Asakawa T, Bernard A, Bjaalie JG, et al. International Brain Initiative: An Innovative Framework for Coordinated Global Brain Research Efforts. Neuron (Cambridge, Mass). 2020;105(5):947. 10.1016/j.neuron.2020.02.022 32135092

[14] Stahl BC, Rainey S, Harris E, Fothergill BT. The role of ethics in data governance of large neuro-ICT projects. J Am Med Inform Assoc. 2018 Aug 1;25(8):1099–107. doi: 10.1093/jamia/ocy040 29767726 PMC6077829

[15] Dove ES. Biobanks, Data Sharing, and the Drive for a Global Privacy Governance Framework. J Law Med Ethics. 2015;43(4):675–89. doi: 10.1111/jlme.12311 26711409

[16] Paik YK, Omenn GS, Uhlen M, Hanash S, Marko-Varga G, Aebersold R, et al. Standard guidelines for the chromosome-centric human proteome project. J Proteome Res. 2012 Apr 6;11(4):2005–13. doi: 10.1021/pr200824a 22443261

[17] Weber K, Otto B, Oesterle H. One Size Does Not Fit All—A Contingency Approach to Data Governance. ACM Journal of Data and Information Quality. 2009 Jun 1;1:Article 4.

[18] Otto B. Organizing Data Governance: Findings from the Telecommunications Industry and Consequences for Large Service Providers. Communications of the Association for Information Systems. 2011;29:3.

[19] Khatri V, Brown CV. Designing data governance. Commun ACM. 2010 Jan 1;53(1):148–52.

[20] Alhassan I, Sammon D, Daly M. Data governance activities: a comparison between scientific and practice-oriented literature. Journal of enterprise information management. 2018;31(2):300–16.

[21] Weill P, Ross JW. IT Governance: How Top Performers Manage IT Decision Rights for Superior Results. Harvard Business Press; 2004. 294 p.

[22] Hammersley M. On ethical principles for social research. International journal of social research methodology. 2015 Jul 4;18(4):433–49.

[23] Lebow RN. The Politics and Ethics of Identity [Internet]. Cambridge: Cambridge University Press; 2012. Available from: 10.1017/CBO9781139226578

[24] Bélanger F, Crossler RE. Privacy in the Digital Age: A Review of Information Privacy Research in Information Systems. MIS quarterly. 2011;35(4):1017–41.

[25] Roskies A. Neuroethics for the New Millenium. Neuron. 2002 Jul 3;35(1):21–3. doi: 10.1016/s0896-6273(02)00763-8 12123605

[26] Salles A, Evers K, Farisco M. The Need for a Conceptual Expansion of Neuroethics. AJOB Neuroscience. 2019 Jul 3;10(3):126–8. doi: 10.1080/21507740.2019.1632972 31329081

[27] Tricco AC, Lillie E, Zarin W, O’Brien KK, Colquhoun H, Levac D, et al. PRISMA Extension for Scoping Reviews (PRISMA-ScR): Checklist and Explanation. Annals of internal medicine. 2018 Oct 2;169(7):467–73. doi: 10.7326/M18-0850 30178033

[28] Grant MJ, Booth A. A typology of reviews: an analysis of 14 review types and associated methodologies. Health information and libraries journal. 2009 Jun;26(2):91–108. doi: 10.1111/j.1471-1842.2009.00848.x 19490148

[29] Sylvester A, Tate M, Johnstone D. Beyond synthesis: re-presenting heterogeneous research literature. Behaviour & information technology. 2013 Dec 1;32(12):1199–215.

[30] White J. PubMed 2.0. Medical reference services quarterly. 2020 Oct 1;39(4):382–7. 33085945 10.1080/02763869.2020.1826228

[31] Bui AAT, Horn JDV. Envisioning the future of ‘big data’ biomedicine. Journal of biomedical informatics. 2017 May;69:115–7. doi: 10.1016/j.jbi.2017.03.017 28366789 PMC5613673

[32] Page MJ, McKenzie JE, Bossuyt PM, Boutron I, Hoffmann TC, Mulrow CD, et al. The PRISMA 2020 statement: An updated guideline for reporting systematic reviews. BMJ British medical journal (International ed) [Internet]. 2021 Mar 29;372. Available from: https://www.narcis.nl/publication/RecordID/oai:pure.amc.nl:publications%2F92617967-0de7-4c29-a234-dc163d6cbf9f 10.1136/bmj.n71 PMC8005924 33782057

[33] NVivo Data Analysis Software [Internet]. 2021 [cited 2021 Nov 11]. Available from: https://www.qsrinternational.com/nvivo-qualitative-data-analysis-software/about/nvivo

[34] Saldaña J. The coding manual for qualitative researchers. London: SAGE Publications Ltd; 2016.

[35] Buller T. The New Ethics of Neuroethics. Cambridge quarterly of healthcare ethics. 2018 Oct;27(4):558–65. doi: 10.1017/S0963180118000087 30720414

[36] Woolley JP. Trust and Justice in Big Data Analytics: Bringing the Philosophical Literature on Trust to Bear on the Ethics of Consent. Philos Technol. 2019;32(1):111–34.

[37] Holmes JH, Elliott TE, Brown JS, Raebel MA, Davidson A, Nelson AF, et al. Clinical research data warehouse governance for distributed research networks in the USA: a systematic review of the literature. J Am Med Inform Assoc. 2014 Aug;21(4):730–6. doi: 10.1136/amiajnl-2013-002370 24682495 PMC4078282

[38] Willison DJ, Trowbridge J, Greiver M, Keshavjee K, Mumford D, Sullivan F. Participatory governance over research in an academic research network: the case of Diabetes Action Canada. BMJ Open. 2019 Apr 20;9(4):e026828. doi: 10.1136/bmjopen-2018-026828 31005936 PMC6500288

[39] Gibbons SMC. Are UK genetic databases governed adequately? A comparative legal analysis. Leg Stud. 2007;27(2):312–42.

[40] Douglas C, Van El C, Radstake M, Van Teeffelen S, Cornel MC. The politics of representation in the governance of emergent ‘secondary use’ biobanks: The case of dried blood spot cards in the Netherlands. Stud Ethics Law Technol [Internet]. 2012;6(1). Available from: https://www.scopus.com/inward/record.uri?eid=2-s2.0-84872853873&doi=10.1515%2f1941-6008.1178&partnerID=40&md5=f4511015bba96f41715721ef92e5dd33

[41] Dove ES, Knoppers BM, Zawati MH. An ethics safe harbor for international genomics research? Genome Med [Internet]. 2013;5(11). Available from: https://www.scopus.com/inward/record.uri?eid=2-s2.0-84888093815&doi=10.1186%2fgm503&partnerID=40&md5=65d9c289741f78f0f147395c2bba0077 24267880 10.1186/gm503 PMC3978721

[42] Kim KK, Browe DK, Logan HC, Holm R, Hack L, Ohno-Machado L. Data governance requirements for distributed clinical research networks: triangulating perspectives of diverse stakeholders. J Am Med Inform Assoc. 2014 Aug;21(4):714–9. doi: 10.1136/amiajnl-2013-002308 24302285 PMC4078279

[43] Vayena E, Blasimme A. Biomedical Big Data: New Models of Control Over Access, Use and Governance. J Bioeth Inq. 2017 Dec;14(4):501–13. doi: 10.1007/s11673-017-9809-6 28983835 PMC5715037

[44] Murtagh MJ, Blell MT, Butters OW, Cowley L, Dove ES, Goodman A, et al. Better governance, better access: practising responsible data sharing in the METADAC governance infrastructure. Hum Genomics. 2018 Apr 26;12(1):24. doi: 10.1186/s40246-018-0154-6 29695297 PMC5918902

[45] Vayena E, Haeusermann T, Adjekum A, Blasimme A. Digital health: meeting the ethical and policy challenges. Swiss Med Wkly. 2018;148:w14571. doi: 10.4414/smw.2018.14571 29376547

[46] Gille F, Vayena E, Blasimme A. Future-proofing biobanks’ governance. Eur J Hum Genet. 2020;28(8):989–96. doi: 10.1038/s41431-020-0646-4 32424324 PMC7468350

[47] McMahon A, Buyx A, Prainsack B. Big Data Governance Needs More Collective Responsibility: The Role of Harm Mitigation in the Governance of Data Use in Medicine and Beyond. Med Law Rev. 2020 Feb 1;28(1):155–82. doi: 10.1093/medlaw/fwz016 31377815

[48] Pavlenko E, Strech D, Langhof H. Implementation of data access and use procedures in clinical data warehouses. A systematic review of literature and publicly available policies. BMC Med Inform Decis Mak. 2020 Jul 11;20(1):157. doi: 10.1186/s12911-020-01177-z 32652989 PMC7353743

[49] Briscoe F, Ajunwa I, Gaddis A, McCormick J. Evolving public views on the value of one’s DNA and expectations for genomic database governance: Results from a national survey. PLoS ONE [Internet]. 2020;15(3). Available from: https://www.scopus.com/inward/record.uri?eid=2-s2.0-85081138917&doi=10.1371%2fjournal.pone.0229044&partnerID=40&md5=853d0db0704738e2ff8c49bbfc52e3aa 32160204 10.1371/journal.pone.0229044 PMC7065739

[50] Evans BJ. Authority of the food and drug administration to require data access and control use rights in the sentinel data network. Food Drug Law J. 2010;65(1):67–112+ii. 24475535

[51] Knoppers BM, Harris JR, Budin-Ljøsne I, Dove ES. A human rights approach to an international code of conduct for genomic and clinical data sharing. Hum Genet. 2014;133(7):895–903. doi: 10.1007/s00439-014-1432-6 24573176 PMC4053599

[52] Rahimzadeh V, Dyke SOM, Knoppers BM. An International Framework for Data Sharing: Moving Forward with the Global Alliance for Genomics and Health. Biopreserv Biobank. 2016 Jun;14(3):256–9. doi: 10.1089/bio.2016.0005 27082668

[53] Reichel J. Alternative Rule-Making within European Bioethics—Necessary and Therefore Legitimate? Tilburg Law Rev. 2016;21(2):169–92.

[54] Sariyar M, Schlünder I. Challenges and Legal Gaps of Genetic Profiling in the Era of Big Data. Front Big Data. 2019;2:40. doi: 10.3389/fdata.2019.00040 33693363 PMC7931923

[55] Schneider G. Health data pools under european policy and data protection law: Research as a new efficiency defence? J Intellect Prop Inf Tech E-Commerce Law. 2020;11(1):49–67.

[56] van Veen EB. Obstacles to European research projects with data and tissue: Solutions and further challenges. Eur J Cancer. 2008;44(10):1438–50. doi: 10.1016/j.ejca.2008.03.011 18440221

[57] Heeney C, Kerr SM. Balancing the local and the universal in maintaining ethical access to a genomics biobank. BMC Med Ethics [Internet]. 2017;18(1). Available from: https://www.scopus.com/inward/record.uri?eid=2-s2.0-85040172871&doi=10.1186%2fs12910-017-0240-7&partnerID=40&md5=6d6f7cf9f251ccea2f3d57db47d41d09 29282045 10.1186/s12910-017-0240-7 PMC5745812

[58] Lin JC, Fan CT, Liao CC, Chen YS. Taiwan Biobank: Making cross-database convergence possible in the Big Data era. GigaScience. 2018;7(1):1–4. doi: 10.1093/gigascience/gix110 29149267 PMC5774504

[59] Brill SB, Moss KO, Prater L. Transformation of the Doctor–Patient Relationship: Big Data, Accountable Care, and Predictive Health Analytics. HEC Forum. 2019;31(4):261–82. doi: 10.1007/s10730-019-09377-5 31209679

[60] Stockdale J, Cassell J, Ford E. “Giving something back”: A systematic review and ethical enquiry into public views on the use of patient data for research in the United Kingdom and the Republic of Ireland [version 2; referees: 2 approved]. Wellcome Open Res [Internet]. 2019;3. Available from: https://www.scopus.com/inward/record.uri?eid=2-s2.0-85063593196&doi=10.12688%2fwellcomeopenres.13531.2&partnerID=40&md5=4764874cf74075f4dcfd74cc9c7e3b71 30854470 10.12688/wellcomeopenres.13531.1 PMC6402072

[61] Ho CWL, Ali J, Caals K. Ensuring trustworthy use of artificial intelligence and big data analytics in health insurance. Bull World Health Organ. 2020 Apr 1;98(4):263–9. doi: 10.2471/BLT.19.234732 32284650 PMC7133481

[62] Taraban R. Limits of Neural Computation in Humans and Machines. Sci Eng Ethics. 2020 Oct;26(5):2547–53. doi: 10.1007/s11948-020-00249-7 32749646

[63] Richesson RL, Horvath MM, Rusincovitch SA. Clinical research informatics and electronic health record data. Yearb Med Inform. 2014;9:215–23. doi: 10.15265/IY-2014-0009 25123746 PMC4287078

[64] Arellano AM, Dai W, Wang S, Jiang X, Ohno-Machado L. Privacy Policy and Technology in Biomedical Data Science. Annu Rev Biomed Data Sci. 2018 Jul;1:115–29. doi: 10.1146/annurev-biodatasci-080917-013416 31058261 PMC6497413

[65] Nellåker C, Alkuraya FS, Baynam G, Bernier RA, Bernier FPJ, Boulanger V, et al. Enabling Global Clinical Collaborations on Identifiable Patient Data: The Minerva Initiative. Front Genet. 2019;10:611. doi: 10.3389/fgene.2019.00611 31417602 PMC6681681

[66] Cormack D, Reid P, Kukutai T. Indigenous data and health: critical approaches to ‘race’/ethnicity and Indigenous data governance. Public Health. 2019 Jul;172:116–8. doi: 10.1016/j.puhe.2019.03.026 31130221

[67] Auray-Blais C, Patenaude J. A biobank management model applicable to biomedical research. BMC Med Ethics [Internet]. 2006;7. Available from: https://www.scopus.com/inward/record.uri?eid=2-s2.0-33744989462&doi=10.1186%2f1472-6939-7-4&partnerID=40&md5=fee515786fd8c105f009ceec6d4877c3 16600040 10.1186/1472-6939-7-4 PMC1475589

[68] Bell JE, Alafuzoff I, Al-Sarraj S, Arzberger T, Bogdanovic N, Budka H, et al. Management of a twenty-first century brain bank: Experience in the BrainNet Europe consortium. Acta Neuropathol. 2008;115(5):497–507. doi: 10.1007/s00401-008-0360-8 18365220

[69] Friedman MJ, Huber BR, Brady CB, Ursano RJ, Benedek DM, Kowall NW, et al. VA’s National PTSD Brain Bank: a National Resource for Research. Curr Psychiatry Rep [Internet]. 2017;19(10). Available from: https://www.scopus.com/inward/record.uri?eid=2-s2.0-85028338814&doi=10.1007%2fs11920-017-0822-6&partnerID=40&md5=602df8c501cda61bc0cacc22468e7cec 10.1007/s11920-017-0822-6 28840457

[70] Chute CG, Beck SA, Fisk TB, Mohr DN. The Enterprise Data Trust at Mayo Clinic: a semantically integrated warehouse of biomedical data. J Am Med Inform Assoc. 2010 Apr;17(2):131–5. doi: 10.1136/jamia.2009.002691 20190054 PMC3000789

[71] Edwards SJL. Protecting privacy interests in brain images: The limits of consent. In: I Know What You’re Think: Brain Imaging and Ment Priv [Internet]. Oxford University Press; 2012. Available from: https://www.scopus.com/inward/record.uri?eid=2-s2.0-84922760541&doi=10.1093%2facprof%3aoso%2f9780199596492.003.0017&partnerID=40&md5=6c209291ea0654ffb80949df7065d4b7

[72] Al-Shahi Salman R, Beller E, Kagan J, Hemminki E, Phillips RS, Savulescu J, et al. Increasing value and reducing waste in biomedical research regulation and management. Lancet. 2014;383(9912):176–85. doi: 10.1016/S0140-6736(13)62297-7 24411646 PMC3952153

[73] Mooser V, Currat C. The lausanne institutional biobank: A new resource to catalyse research in personalised medicine and pharmaceutical sciences. Swiss Med Wkly [Internet]. 2014;144. Available from: https://www.scopus.com/inward/record.uri?eid=2-s2.0-84928970051&doi=10.4414%2fsmw.2014.14033&partnerID=40&md5=a71af9ae782ee42b908d5daf06e433bd 25474562 10.4414/smw.2014.14033

[74] Kaye J, Briceño Moraia L, Curren L, Bell J, Mitchell C, Soini S, et al. Consent for Biobanking: The Legal Frameworks of Countries in the BioSHaRE-EU Project. Biopreservation Biobanking. 2016;14(3):195–200. doi: 10.1089/bio.2015.0123 27145287 PMC5967579

[75] Kaye J, Terry SF, Juengst E, Coy S, Harris JR, Chalmers D, et al. Including all voices in international datasharing governance. Hum Genomics [Internet]. 2018;12(1). Available from: https://www.scopus.com/inward/record.uri?eid=2-s2.0-85054512218&doi=10.1186%2fs40246-018-0143-9&partnerID=40&md5=a0ded4b410077789f32a31785fde1ad0 10.1186/s40246-018-0143-9 PMC5842530 29514717

[76] Mittelstadt BD, Floridi L. The Ethics of Big Data: Current and Foreseeable Issues in Biomedical Contexts. Sci Eng Ethics. 2016;22(2):303–41. doi: 10.1007/s11948-015-9652-2 26002496

[77] Sutherland GT, Sheedy D, Stevens J, McCrossin T, Smith CC, van Roijen M, et al. The NSW brain tissue resource centre: Banking for alcohol and major neuropsychiatric disorders research. Alcohol. 2016;52:33–9. doi: 10.1016/j.alcohol.2016.02.005 27139235 PMC4855871

[78] Woolley JP, McGowan ML, Teare HJA, Coathup V, Fishman JR, Settersten RA Jr, et al. Citizen science or scientific citizenship? Disentangling the uses of public engagement rhetoric in national research initiatives Donna Dickenson, Sandra Soo-Jin Lee, and Michael Morrison. BMC Med Ethics [Internet]. 2016;17(1). Available from: https://www.scopus.com/inward/record.uri?eid=2-s2.0-84971624242&doi=10.1186%2fs12910-016-0117-1&partnerID=40&md5=aacd51bfda96dfe45c68852d0b538f9d 10.1186/s12910-016-0117-1 PMC4893207 27260081

[79] Ballantyne A, Style R. Health data research in New Zealand: updating the ethical governance framework. N Z Med J. 2017 Oct 27;130(1464):64–71. 29073658

[80] Ho CH. Challenges of the EU general data protection regulation for biobanking and scientific research. J Law Inf Sci. 2017;25(1):84–103.

[81] Vezyridis P, Timmons S. Understanding the care.data conundrum: New information flows for economic growth. Big Data Soc [Internet]. 2017;4(1). Available from: https://www.scopus.com/inward/record.uri?eid=2-s2.0-85032789524&doi=10.1177%2f2053951716688490&partnerID=40&md5=91ad1cef4d8d6016388a89f2fd47e7a9

[82] Dankar FK, Ptitsyn A, Dankar SK. The development of large-scale de-identified biomedical databases in the age of genomics-principles and challenges. Hum Genomics. 2018 Apr 10;12(1):19. doi: 10.1186/s40246-018-0147-5 29636096 PMC5894154

[83] Dorey M C, Baumann H, Biller-Andorno N. Patient data and patient rights: Swiss healthcare stakeholders’ ethical awareness regarding large patient data sets—A qualitative study. BMC Med Ethics [Internet]. 2018;19(1). Available from: https://www.scopus.com/inward/record.uri?eid=2-s2.0-85043249366&doi=10.1186%2fs12910-018-0261-x&partnerID=40&md5=9973c60023850c5a7d741a48ba086764 10.1186/s12910-018-0261-x PMC5842517 29514635

[84] Staccini P, Lau AYS. Findings from 2017 on Consumer Health Informatics and Education: Health Data Access and Sharing. Yearb Med Inform. 2018;27(1):163–9. doi: 10.1055/s-0038-1641218 30157519 PMC6115219

[85] Beier K, Schweda M, Schicktanz S. Taking patient involvement seriously: A critical ethical analysis of participatory approaches in data-intensive medical research. BMC Med Informatics Decis Mak [Internet]. 2019;19(1). Available from: https://www.scopus.com/inward/record.uri?eid=2-s2.0-85065299747&doi=10.1186%2fs12911-019-0799-7&partnerID=40&md5=ce2d8a3ec2f88254a8cced06ecd98368 31023321 10.1186/s12911-019-0799-7 PMC6482526

[86] Bot BM, Wilbanks JT, Mangravite LM. Assessing the consequences of decentralizing biomedical research. Big Data Soc [Internet]. 2019;6(1). Available from: https://www.scopus.com/inward/record.uri?eid=2-s2.0-85074144728&doi=10.1177%2f2053951719853858&partnerID=40&md5=e83258a1929a0a95fe694ccba06b130a

[87] Lefaivre S, Behan B, Vaccarino A, Evans K, Dharsee M, Gee T, et al. Big Data Needs Big Governance: Best Practices From Brain-CODE, the Ontario-Brain Institute’s Neuroinformatics Platform. Front Genet. 2019;10:191. doi: 10.3389/fgene.2019.00191 30984233 PMC6450217

[88] Erikainen S, Friesen P, Rand L, Jongsma K, Dunn M, Sorbie A, et al. Public involvement in the governance of population-level biomedical research: Unresolved questions and future directions. J Med Ethics [Internet]. 2020; Available from: https://www.scopus.com/inward/record.uri?eid=2-s2.0-85092797300&doi=10.1136%2fmedethics-2020-106530&partnerID=40&md5=88cdf549faca6b0e1a1f4b074650b678 33023977 10.1136/medethics-2020-106530

[89] Milne R, Brayne C. We need to think about data governance for dementia research in a digital era. Alzheimers Res Ther. 2020 Jan 31;12(1):17. doi: 10.1186/s13195-020-0584-y 32005135 PMC6995068

[90] Shabani M. The Data Governance Act and the EU’s move towards facilitating data sharing. Molecular Systems Biology [Internet]. 2021 Mar;17(3). Available from: https://search.proquest.com/scholarly-journals/data-governance-act-eus-move-towards-facilitating/docview/2506672179/se-2?accountid=10472 doi: 10.15252/msb.202110229 33755313 PMC7985992

[91] Prokosch HU, Acker T, Bernarding J, Binder H, Boeker M, Boerries M, et al. MIRACUM: Medical Informatics in Research and Care in University Medicine. Methods Inf Med. 2018;57(S 01):e82–91. doi: 10.3414/ME17-02-0025 30016814 PMC6178200

[92] Wulff A, Haarbrandt B, Marschollek M. Clinical Knowledge Governance Framework for Nationwide Data Infrastructure Projects…"Biomedical Meets eHealth ‘From Sensors to Decisions,’—papers from the 12th eHealth conference, held in Vienna, Austria, May 8–9, 2018. Studies in Health Technology & Informatics. 2018 May;248:196–200. 29726437

[93] Wilson RC, Butters OW, Avraam D, Baker J, Tedds JA, Turner A, et al. DataSHIELD—New directions and dimensions. Data Sci J [Internet]. 2017;16. Available from: https://www.scopus.com/inward/record.uri?eid=2-s2.0-85020536872&doi=10.5334%2fdsj-2017-021&partnerID=40&md5=85346c992bd2f72e9f5a3d8e758d1526

[94] Bollinger JM, Zuk PD, Majumder MA, Versalovic E, Villanueva AG, Hsu RL, et al. What is a Medical Information Commons? J Law Med Ethics. 2019;47(1):41–50. doi: 10.1177/1073110519840483 30994065 PMC6730652

[95] Parciak M, Bender T, Sax U, Bauer CR. Applying FAIRness: Redesigning a Biomedical Informatics Research Data Management Pipeline. Methods Inf Med. 2019 Dec;58(6):229–34. doi: 10.1055/s-0040-1709158 32349157

[96] Parciak M, Bauer C, Bender T, Lodahl R, Schreiweis B, Tute E, et al. Provenance Solutions for Medical Research in Heterogeneous IT-Infrastructure: An Implementation Roadmap. Stud Health Technol Inform. 2019 Aug 21;264:298–302. doi: 10.3233/SHTI190231 31437933

[97] Simell BA, Törnwall OM, Hämäläinen I, Wichmann HE, Anton G, Brennan P, et al. Transnational access to large prospective cohorts in Europe: Current trends and unmet needs. New Biotechnol. 2019;49:98–103. doi: 10.1016/j.nbt.2018.10.001 30342241

[98] Deshpande P, Rasin A, Furst J, Raicu D, Antani S. DiiS: A biomedical data access framework for aiding data driven research supporting FAIR principles. Data [Internet]. 2019;4(2). Available from: https://www.scopus.com/inward/record.uri?eid=2-s2.0-85070852759&doi=10.3390%2fdata4020054&partnerID=40&md5=07fd84565ecef7135e3bef58ccb7e08d

[99] Tosetti P, Hicks RR, Theriault E, Phillips A, Koroshetz W, Draghia-Akli R. Toward an international initiative for traumatic brain injury research. J Neurotrauma. 2013;30(14):1211–22. doi: 10.1089/neu.2013.2896 23731282 PMC3713440

[100] Ward HJT. Privacy and governance implications of wider societal uses of brain imaging data. Cortex. 2011;47(10):1263–5. doi: 10.1016/j.cortex.2011.04.016 21620388

[101] Andreu-Perez J., Poon C. C. Y., Merrifield R. D., Wong S. T. C., Yang G. Big Data for Health. IEEE Journal of Biomedical and Health Informatics. 2015 Jul;19(4):1193–208. doi: 10.1109/JBHI.2015.2450362 26173222

[102] Lopez MH, Holve E, Sarkar IN, Segal C. Building the informatics infrastructure for comparative effectiveness research (CER): a review of the literature. Med Care. 2012 Jul;50 Suppl:S38–48. doi: 10.1097/MLR.0b013e318259becd 22692258

[103] Haarbrandt B, Schreiweis B, Rey S, Sax U, Scheithauer S, Rienhoff O, et al. HiGHmed—An Open Platform Approach to Enhance Care and Research across Institutional Boundaries. Methods Inf Med. 2018;57(S 01):e66–81. doi: 10.3414/ME18-02-0002 30016813 PMC6193407

[104] Dagliati A, Malovini A, Tibollo V, Bellazzi R. Health informatics and EHR to support clinical research in the COVID-19 pandemic: an overview. Brief Bioinform. 2021 Mar 22;22(2):812–22. doi: 10.1093/bib/bbaa418 33454728 PMC7929411

[105] Tempini N, Leonelli S. Concealment and discovery: The role of information security in biomedical data re-use. Soc Stud Sci. 2018;48(5):663–90. doi: 10.1177/0306312718804875 30322372 PMC6193209

[106] Shah N, Coathup V, Teare H, Forgie I, Giordano GN, Hansen TH, et al. Sharing data for future research-engaging participants’ views about data governance beyond the original project: a DIRECT Study. Genet Med. 2019 May;21(5):1131–8. doi: 10.1038/s41436-018-0299-7 30262927

[107] Morrison M, Mourby M, Gowans H, Coy S, Kaye J. Governance of research consortia: challenges of implementing Responsible Research and Innovation within Europe. Life Sci Soc Policy [Internet]. 2020;16(1). Available from: https://www.scopus.com/inward/record.uri?eid=2-s2.0-85095984089&doi=10.1186%2fs40504-020-00109-z&partnerID=40&md5=8b12f647e53a1f21460beb2104c6e938 10.1186/s40504-020-00109-z PMC7667809 33190636

[108] Ballantyne A, Stewart C. Big Data and Public-Private Partnerships in Healthcare and Research: The Application of an Ethics Framework for Big Data in Health and Research. Asian Bioeth Rev. 2019;11(3):315–26. doi: 10.1007/s41649-019-00100-7 33717319 PMC7747238

[109] Nicol D, Critchley C, McWhirter R, Whitton T. Understanding public reactions to commercialization of biobanks and use of biobank resources. Soc Sci Med. 2016;162:79–87. doi: 10.1016/j.socscimed.2016.06.028 27343817

[110] Dzobo K, Adotey S, Thomford NE, Dzobo W. Integrating Artificial and Human Intelligence: A Partnership for Responsible Innovation in Biomedical Engineering and Medicine. OMICS J Integr Biol. 2020;24(5):247–63. doi: 10.1089/omi.2019.0038 31313972

[111] Bossert S, Kahrass H, Strech D. The public’s awareness of and attitude toward research biobanks—A regional German survey. Front Genet [Internet]. 2018;9(MAY). Available from: https://www.scopus.com/inward/record.uri?eid=2-s2.0-85047506782&doi=10.3389%2ffgene.2018.00190&partnerID=40&md5=515f764dc75c043061f8917a81f27e46 10.3389/fgene.2018.00190 PMC5977155 29881399

[112] Manion FJ, Robbins RJ, Weems WA, Crowley RS. Security and privacy requirements for a multi-institutional cancer research data grid: An interview-based study. BMC Med Informatics Decis Mak [Internet]. 2009;9(1). Available from: https://www.scopus.com/inward/record.uri?eid=2-s2.0-67949124695&doi=10.1186%2f1472-6947-9-31&partnerID=40&md5=df6aad301fa8a4f981f62b3741b2bf81 19527521 10.1186/1472-6947-9-31 PMC2709611

[113] Ireni-Saban L. Genomics governance in the United States and the United Kingdom. European J Comp Law Gov. 2014;1(3):244–6.

[114] Salter B, Salter C. Controlling new knowledge: Genomic science, governance and the politics of bioinformatics. Soc Stud Sci. 2017;47(2):263–87. doi: 10.1177/0306312716681210 28056721 PMC5405805

[115] Langhof H, Kahrass H, Illig T, Jahns R, Strech D. Current practices for access, compensation, and prioritization in biobanks. Results from an interview study. Eur J Hum Genet. 2018;26(11):1572–81. doi: 10.1038/s41431-018-0228-x 30089824 PMC6189200

[116] Willems SM, Abeln S, Feenstra KA, de Bree R, van der Poel EF, Baatenburg de Jong RJ, et al. The potential use of big data in oncology. Oral Oncol. 2019;98:8–12. doi: 10.1016/j.oraloncology.2019.09.003 31521885

[117] Zúñiga-Fajuri A, Miranda LV, Miralles DZ, Venegas RS. Chapter Seven—Neurorights in Chile: Between neuroscience and legal science. In: Hevia M, editor. Developments in Neuroethics and Bioethics [Internet]. Academic Press; 2021 [cited 2022 Apr 15]. p. 165–79. (Regulating Neuroscience: Transnational Legal Challenges; vol. 4). Available from: https://www.sciencedirect.com/science/article/pii/S2589295921000059

[118] Bublitz JC. Novel Neurorights: From Nonsense to Substance. Neuroethics. 2022 Feb 8;15(1):7. doi: 10.1007/s12152-022-09481-3 35154507 PMC8821782

[119] Malgieri G. Data protection and research: A vital challenge in the era of COVID-19 pandemic. Computer Law & Security Review. 2020 Jul;37:105431.

[120] Ducato R. Data protection, scientific research, and the role of information. Computer Law & Security Review. 2020 Jul 1;37:105412.

[121] Bloomrosen M, Berner ES. Findings from the 2017 Yearbook Section on Health Information Management. Yearb Med Inform. 2017;26(1):78–83. doi: 10.15265/IY-2017-025 29063540 PMC6239241

[122] Oliveira JL, Trifan A, Bastião Silva LA. EMIF Catalogue: A collaborative platform for sharing and reusing biomedical data. Int J Med Inform. 2019 Jun;126:35–45. doi: 10.1016/j.ijmedinf.2019.02.006 31029262

[123] Knoppers BM, Abdul-Rahman MH, Bédard K. Genomic Databases and International Collaboration. King’s Law Journal. 2007 Jan 1;18(2):291–311.

[124] Floridi L. The Fourth Revolution: How the Infosphere is Reshaping Human Reality. OUP Oxford; 2014. 265 p.

[125] Bawden D, Robinson L. “The dearest of our possessions”: Applying Floridi’s information privacy concept in models of information behavior and information literacy. Journal of the Association for Information Science and Technology. 2020;71(9):1030–43.

