# Supplementary material for: The ethical and legal landscape of brain data governance
Source: PLoS One. 2022 Dec 29;17(12):e0273473. doi: 10.1371/journal.pone.0273473 (PMC9799320; doi:10.1371/journal.pone.0273473)
Supplement: S1 File — (DOCX) [file pone.0273473.s002.docx]

1. Auray-Blais C, Patenaude J. A biobank management model applicable to biomedical research. BMC Med Ethics [Internet]. 2006;7. Available from: https://www.scopus.com/inward/record.uri?eid=2-s2.0-33744989462&doi=10.1186%2f1472-6939-7-4&partnerID=40&md5=fee515786fd8c105f009ceec6d4877c3

2. Knoppers BM, Harris JR, Budin-Ljøsne I, Dove ES. A human rights approach to an international code of conduct for genomic and clinical data sharing. Hum Genet. 2014;133(7):895–903.

3. Reichel J. Alternative Rule-Making within European Bioethics - Necessary and Therefore Legitimate? Tilburg Law Rev. 2016;21(2):169–92.

4. Dove ES, Knoppers BM, Zawati MH. An ethics safe harbor for international genomics research? Genome Med [Internet]. 2013;5(11). Available from: https://www.scopus.com/inward/record.uri?eid=2-s2.0-84888093815&doi=10.1186%2fgm503&partnerID=40&md5=65d9c289741f78f0f147395c2bba0077

5. Rahimzadeh V, Dyke SOM, Knoppers BM. An International Framework for Data Sharing: Moving Forward with the Global Alliance for Genomics and Health. Biopreserv Biobank. 2016 Jun;14(3):256–9.

6. Parciak M, Bender T, Sax U, Bauer CR. Applying FAIRness: Redesigning a Biomedical Informatics Research Data Management Pipeline. Methods Inf Med. 2019 Dec;58(6):229–34.

7. Gibbons SMC. Are UK genetic databases governed adequately? A comparative legal analysis. Leg Stud. 2007;27(2):312–42.

8. Bot BM, Wilbanks JT, Mangravite LM. Assessing the consequences of decentralizing biomedical research. Big Data Soc [Internet]. 2019;6(1). Available from: https://www.scopus.com/inward/record.uri?eid=2-s2.0-85074144728&doi=10.1177%2f2053951719853858&partnerID=40&md5=e83258a1929a0a95fe694ccba06b130a

9. Evans BJ. Authority of the food and drug administration to require data access and control use rights in the sentinel data network. Food Drug Law J. 2010;65(1):67-112+ii.

10. Heeney C, Kerr SM. Balancing the local and the universal in maintaining ethical access to a genomics biobank. BMC Med Ethics [Internet]. 2017;18(1). Available from: https://www.scopus.com/inward/record.uri?eid=2-s2.0-85040172871&doi=10.1186%2fs12910-017-0240-7&partnerID=40&md5=6d6f7cf9f251ccea2f3d57db47d41d09

11. Murtagh MJ, Blell MT, Butters OW, Cowley L, Dove ES, Goodman A, et al. Better governance, better access: practising responsible data sharing in the METADAC governance infrastructure. Hum Genomics. 2018 Apr 26;12(1):24.

12. Kellmeyer P. Big Brain Data: On the Responsible Use of Brain Data from Clinical and Consumer-Directed Neurotechnological Devices [Internet]. 2018. Available from: https://www.scopus.com/inward/record.uri?eid=2-s2.0-85047113952&doi=10.1007%2fs12152-018-9371-x&partnerID=40&md5=60e569f21f0301e42a6d94e76fe75295

13. Ballantyne A, Stewart C. Big Data and Public-Private Partnerships in Healthcare and Research: The Application of an Ethics Framework for Big Data in Health and Research. Asian Bioeth Rev. 2019;11(3):315–26.

14. J. Andreu-Perez, C. C. Y. Poon, R. D. Merrifield, S. T. C. Wong, G. Yang. Big Data for Health. IEEE Journal of Biomedical and Health Informatics. 2015 Jul;19(4):1193–208.

15. McMahon A, Buyx A, Prainsack B. Big Data Governance Needs More Collective Responsibility: The Role of Harm Mitigation in the Governance of Data Use in Medicine and Beyond. Med Law Rev. 2020 Feb 1;28(1):155–82.

16. Lefaivre S, Behan B, Vaccarino A, Evans K, Dharsee M, Gee T, et al. Big Data Needs Big Governance: Best Practices From Brain-CODE, the Ontario-Brain Institute’s Neuroinformatics Platform. Front Genet. 2019;10:191.

17. Dove ES. Biobanks, Data Sharing, and the Drive for a Global Privacy Governance Framework. J Law Med Ethics. 2015;43(4):675–89.

18. Vayena E, Blasimme A. Biomedical Big Data: New Models of Control Over Access, Use and Governance. J Bioeth Inq. 2017 Dec;14(4):501–13.

19. Lopez MH, Holve E, Sarkar IN, Segal C. Building the informatics infrastructure for comparative effectiveness research (CER): a review of the literature. Med Care. 2012 Jul;50 Suppl:S38-48.

20. Sariyar M, Schlünder I. Challenges and Legal Gaps of Genetic Profiling in the Era of Big Data. Front Big Data. 2019;2:40.

21. Ho C-H. Challenges of the EU general data protection regulation for biobanking and scientific research. J Law Inf Sci. 2017;25(1):84–103.

22. Woolley JP, McGowan ML, Teare HJA, Coathup V, Fishman JR, Settersten RA Jr, et al. Citizen science or scientific citizenship? Disentangling the uses of public engagement rhetoric in national research initiatives Donna Dickenson, Sandra Soo-Jin Lee, and Michael Morrison. BMC Med Ethics [Internet]. 2016;17(1). Available from: https://www.scopus.com/inward/record.uri?eid=2-s2.0-84971624242&doi=10.1186%2fs12910-016-0117-1&partnerID=40&md5=aacd51bfda96dfe45c68852d0b538f9d

23. Wulff A, Haarbrandt B, Marschollek M. Clinical Knowledge Governance Framework for Nationwide Data Infrastructure Projects..."Biomedical Meets eHealth ‘From Sensors to Decisions,’ -- papers from the 12th eHealth conference, held in Vienna, Austria, May 8-9, 2018. Studies in Health Technology & Informatics. 2018 May;248:196–200.

24. Holmes JH, Elliott TE, Brown JS, Raebel MA, Davidson A, Nelson AF, et al. Clinical research data warehouse governance for distributed research networks in the USA: a systematic review of the literature. J Am Med Inform Assoc. 2014 Aug;21(4):730–6.

25. Richesson RL, Horvath MM, Rusincovitch SA. Clinical research informatics and electronic health record data. Yearb Med Inform. 2014;9:215–23.

26. Tempini N, Leonelli S. Concealment and discovery: The role of information security in biomedical data re-use. Soc Stud Sci. 2018;48(5):663–90.

27. Kaye J, Briceño Moraia L, Curren L, Bell J, Mitchell C, Soini S, et al. Consent for Biobanking: The Legal Frameworks of Countries in the BioSHaRE-EU Project. Biopreservation Biobanking. 2016;14(3):195–200.

28. Salter B, Salter C. Controlling new knowledge: Genomic science, governance and the politics of bioinformatics. Soc Stud Sci. 2017;47(2):263–87.

29. Langhof H, Kahrass H, Illig T, Jahns R, Strech D. Current practices for access, compensation, and prioritization in biobanks. Results from an interview study. Eur J Hum Genet. 2018;26(11):1572–81.

30. Kim KK, Browe DK, Logan HC, Holm R, Hack L, Ohno-Machado L. Data governance requirements for distributed clinical research networks: triangulating perspectives of diverse stakeholders. J Am Med Inform Assoc. 2014 Aug;21(4):714–9.

31. Wilson RC, Butters OW, Avraam D, Baker J, Tedds JA, Turner A, et al. DataSHIELD - New directions and dimensions. Data Sci J [Internet]. 2017;16. Available from: https://www.scopus.com/inward/record.uri?eid=2-s2.0-85020536872&doi=10.5334%2fdsj-2017-021&partnerID=40&md5=85346c992bd2f72e9f5a3d8e758d1526

32. Vayena E, Haeusermann T, Adjekum A, Blasimme A. Digital health: meeting the ethical and policy challenges. Swiss Med Wkly. 2018;148:w14571.

33. Deshpande P, Rasin A, Furst J, Raicu D, Antani S. DiiS: A biomedical data access framework for aiding data driven research supporting FAIR principles. Data [Internet]. 2019;4(2). Available from: https://www.scopus.com/inward/record.uri?eid=2-s2.0-85070852759&doi=10.3390%2fdata4020054&partnerID=40&md5=07fd84565ecef7135e3bef58ccb7e08d

34. Oliveira JL, Trifan A, Bastião Silva LA. EMIF Catalogue: A collaborative platform for sharing and reusing biomedical data. Int J Med Inform. 2019 Jun;126:35–45.

35. Nellåker C, Alkuraya FS, Baynam G, Bernier RA, Bernier FPJ, Boulanger V, et al. Enabling Global Clinical Collaborations on Identifiable Patient Data: The Minerva Initiative. Front Genet. 2019;10:611.

36. Ho CWL, Ali J, Caals K. Ensuring trustworthy use of artificial intelligence and big data analytics in health insurance. Bull World Health Organ. 2020 Apr 1;98(4):263–9.

37. Briscoe F, Ajunwa I, Gaddis A, McCormick J. Evolving public views on the value of one’s DNA and expectations for genomic database governance: Results from a national survey. PLoS ONE [Internet]. 2020;15(3). Available from: https://www.scopus.com/inward/record.uri?eid=2-s2.0-85081138917&doi=10.1371%2fjournal.pone.0229044&partnerID=40&md5=853d0db0704738e2ff8c49bbfc52e3aa

38. Staccini P, Lau AYS. Findings from 2017 on Consumer Health Informatics and Education: Health Data Access and Sharing. Yearb Med Inform. 2018;27(1):163–9.

39. Bloomrosen M, Berner ES. Findings from the 2017 Yearbook Section on Health Information Management. Yearb Med Inform. 2017;26(1):78–83.

40. Gille F, Vayena E, Blasimme A. Future-proofing biobanks’ governance. Eur J Hum Genet. 2020;28(8):989–96.

41. Ireni-Saban L. Genomics governance in the United States and the United Kingdom. European J Comp Law Gov. 2014;1(3):244–6.

42. Stockdale J, Cassell J, Ford E. “Giving something back”: A systematic review and ethical enquiry into public views on the use of patient data for research in the United Kingdom and the Republic of Ireland [version 2; referees: 2 approved]. Wellcome Open Res [Internet]. 2019;3. Available from: https://www.scopus.com/inward/record.uri?eid=2-s2.0-85063593196&doi=10.12688%2fwellcomeopenres.13531.2&partnerID=40&md5=4764874cf74075f4dcfd74cc9c7e3b71

43. Morrison M, Mourby M, Gowans H, Coy S, Kaye J. Governance of research consortia: challenges of implementing Responsible Research and Innovation within Europe. Life Sci Soc Policy [Internet]. 2020;16(1). Available from: https://www.scopus.com/inward/record.uri?eid=2-s2.0-85095984089&doi=10.1186%2fs40504-020-00109-z&partnerID=40&md5=8b12f647e53a1f21460beb2104c6e938

44. Schneider G. Health data pools under european policy and data protection law: Research as a new efficiency defence? J Intellect Prop Inf Tech E-Commerce Law. 2020;11(1):49–67.

45. Ballantyne A, Style R. Health data research in New Zealand: updating the ethical governance framework. N Z Med J. 2017 Oct 27;130(1464):64–71.

46. Dagliati A, Malovini A, Tibollo V, Bellazzi R. Health informatics and EHR to support clinical research in the COVID-19 pandemic: an overview. Brief Bioinform. 2021 Mar 22;22(2):812–22.

47. Haarbrandt B, Schreiweis B, Rey S, Sax U, Scheithauer S, Rienhoff O, et al. HiGHmed - An Open Platform Approach to Enhance Care and Research across Institutional Boundaries. Methods Inf Med. 2018;57(S 01):e66–81.

48. Pavlenko E, Strech D, Langhof H. Implementation of data access and use procedures in clinical data warehouses. A systematic review of literature and publicly available policies. BMC Med Inform Decis Mak. 2020 Jul 11;20(1):157.

49. Kaye J, Terry SF, Juengst E, Coy S, Harris JR, Chalmers D, et al. Including all voices in international datasharing governance. Hum Genomics [Internet]. 2018;12(1). Available from: https://www.scopus.com/inward/record.uri?eid=2-s2.0-85054512218&doi=10.1186%2fs40246-018-0143-9&partnerID=40&md5=a0ded4b410077789f32a31785fde1ad0

50. Al-Shahi Salman R, Beller E, Kagan J, Hemminki E, Phillips RS, Savulescu J, et al. Increasing value and reducing waste in biomedical research regulation and management. Lancet. 2014;383(9912):176–85.

51. Cormack D, Reid P, Kukutai T. Indigenous data and health: critical approaches to ’race’/ethnicity and Indigenous data governance. Public Health. 2019 Jul;172:116–8.

52. Dzobo K, Adotey S, Thomford NE, Dzobo W. Integrating Artificial and Human Intelligence: A Partnership for Responsible Innovation in Biomedical Engineering and Medicine. OMICS J Integr Biol. 2020;24(5):247–63.

53. Taraban R. Limits of Neural Computation in Humans and Machines. Sci Eng Ethics. 2020 Oct;26(5):2547–53.

54. Bell JE, Alafuzoff I, Al-Sarraj S, Arzberger T, Bogdanovic N, Budka H, et al. Management of a twenty-first century brain bank: Experience in the BrainNet Europe consortium. Acta Neuropathol. 2008;115(5):497–507.

55. Prokosch H-U, Acker T, Bernarding J, Binder H, Boeker M, Boerries M, et al. MIRACUM: Medical Informatics in Research and Care in University Medicine. Methods Inf Med. 2018;57(S 01):e82–91.

56. van Veen E-B. Obstacles to European research projects with data and tissue: Solutions and further challenges. Eur J Cancer. 2008;44(10):1438–50.

57. Willison DJ, Trowbridge J, Greiver M, Keshavjee K, Mumford D, Sullivan F. Participatory governance over research in an academic research network: the case of Diabetes Action Canada. BMJ Open. 2019 Apr 20;9(4):e026828.

58. Dorey M C, Baumann H, Biller-Andorno N. Patient data and patient rights: Swiss healthcare stakeholders’ ethical awareness regarding large patient data sets - A qualitative study. BMC Med Ethics [Internet]. 2018;19(1). Available from: https://www.scopus.com/inward/record.uri?eid=2-s2.0-85043249366&doi=10.1186%2fs12910-018-0261-x&partnerID=40&md5=9973c60023850c5a7d741a48ba086764

59. Ward HJT. Privacy and governance implications of wider societal uses of brain imaging data. Cortex. 2011;47(10):1263–5.

60. Arellano AM, Dai W, Wang S, Jiang X, Ohno-Machado L. Privacy Policy and Technology in Biomedical Data Science. Annu Rev Biomed Data Sci. 2018 Jul;1:115–29.

61. Edwards SJL. Protecting privacy interests in brain images: The limits of consent. In: I Know What You’re Think: Brain Imaging and Ment Priv [Internet]. Oxford University Press; 2012. Available from: https://www.scopus.com/inward/record.uri?eid=2-s2.0-84922760541&doi=10.1093%2facprof%3aoso%2f9780199596492.003.0017&partnerID=40&md5=6c209291ea0654ffb80949df7065d4b7

62. Parciak M, Bauer C, Bender T, Lodahl R, Schreiweis B, Tute E, et al. Provenance Solutions for Medical Research in Heterogeneous IT-Infrastructure: An Implementation Roadmap. Stud Health Technol Inform. 2019 Aug 21;264:298–302.

63. Erikainen S, Friesen P, Rand L, Jongsma K, Dunn M, Sorbie A, et al. Public involvement in the governance of population-level biomedical research: Unresolved questions and future directions. J Med Ethics [Internet]. 2020; Available from: https://www.scopus.com/inward/record.uri?eid=2-s2.0-85092797300&doi=10.1136%2fmedethics-2020-106530&partnerID=40&md5=88cdf549faca6b0e1a1f4b074650b678

64. Fothergill BT, Knight W, Stahl BC, Ulnicane I. Responsible Data Governance of Neuroscience Big Data. Front Neuroinform. 2019;13:28.

65. Manion FJ, Robbins RJ, Weems WA, Crowley RS. Security and privacy requirements for a multi-institutional cancer research data grid: An interview-based study. BMC Med Informatics Decis Mak [Internet]. 2009;9(1). Available from: https://www.scopus.com/inward/record.uri?eid=2-s2.0-67949124695&doi=10.1186%2f1472-6947-9-31&partnerID=40&md5=df6aad301fa8a4f981f62b3741b2bf81

66. Shah N, Coathup V, Teare H, Forgie I, Giordano GN, Hansen TH, et al. Sharing data for future research-engaging participants’ views about data governance beyond the original project: a DIRECT Study. Genet Med. 2019 May;21(5):1131–8.

67. Paik Y-K, Omenn GS, Uhlen M, Hanash S, Marko-Varga G, Aebersold R, et al. Standard guidelines for the chromosome-centric human proteome project. J Proteome Res. 2012 Apr 6;11(4):2005–13.

68. Lin J-C, Fan C-T, Liao C-C, Chen Y-S. Taiwan Biobank: Making cross-database convergence possible in the Big Data era. GigaScience. 2018;7(1):1–4.

69. Beier K, Schweda M, Schicktanz S. Taking patient involvement seriously: A critical ethical analysis of participatory approaches in data-intensive medical research. BMC Med Informatics Decis Mak [Internet]. 2019;19(1). Available from: https://www.scopus.com/inward/record.uri?eid=2-s2.0-85065299747&doi=10.1186%2fs12911-019-0799-7&partnerID=40&md5=ce2d8a3ec2f88254a8cced06ecd98368

70. Shabani M. The Data Governance Act and the EU’s move towards facilitating data sharing. Mol Syst Biol. 2021 Mar;17(3):e10229.

71. Dankar FK, Ptitsyn A, Dankar SK. The development of large-scale de-identified biomedical databases in the age of genomics-principles and challenges. Hum Genomics. 2018 Apr 10;12(1):19.

72. Chute CG, Beck SA, Fisk TB, Mohr DN. The Enterprise Data Trust at Mayo Clinic: a semantically integrated warehouse of biomedical data. J Am Med Inform Assoc. 2010 Apr;17(2):131–5.

73. Mittelstadt BD, Floridi L. The Ethics of Big Data: Current and Foreseeable Issues in Biomedical Contexts. Sci Eng Ethics. 2016;22(2):303–41.

74. Teare HJA, De Masi F, Banasik K, Barnett A, Herrgard S, Jablonka B, et al. The governance structure for data access in the DIRECT consortium: An innovative medicines initiative (IMI) project. Life Sci Soc Policy [Internet]. 2018;14(1). Available from: https://www.scopus.com/inward/record.uri?eid=2-s2.0-85053157053&doi=10.1186%2fs40504-018-0083-0&partnerID=40&md5=362afb08d7c25dc6304d9ec4fca49128

75. Mooser V, Currat C. The lausanne institutional biobank: A new resource to catalyse research in personalised medicine and pharmaceutical sciences. Swiss Med Wkly [Internet]. 2014;144. Available from: https://www.scopus.com/inward/record.uri?eid=2-s2.0-84928970051&doi=10.4414%2fsmw.2014.14033&partnerID=40&md5=a71af9ae782ee42b908d5daf06e433bd

76. Sutherland GT, Sheedy D, Stevens J, McCrossin T, Smith CC, van Roijen M, et al. The NSW brain tissue resource centre: Banking for alcohol and major neuropsychiatric disorders research. Alcohol. 2016;52:33–9.

77. Douglas C, Van El C, Radstake M, Van Teeffelen S, Cornel MC. The politics of representation in the governance of emergent ‘secondary use’ biobanks: The case of dried blood spot cards in the Netherlands. Stud Ethics Law Technol [Internet]. 2012;6(1). Available from: https://www.scopus.com/inward/record.uri?eid=2-s2.0-84872853873&doi=10.1515%2f1941-6008.1178&partnerID=40&md5=f4511015bba96f41715721ef92e5dd33

78. Willems SM, Abeln S, Feenstra KA, de Bree R, van der Poel EF, Baatenburg de Jong RJ, et al. The potential use of big data in oncology. Oral Oncol. 2019;98:8–12.

79. Bossert S, Kahrass H, Strech D. The public’s awareness of and attitude toward research biobanks - A regional German survey. Front Genet [Internet]. 2018;9(MAY). Available from: https://www.scopus.com/inward/record.uri?eid=2-s2.0-85047506782&doi=10.3389%2ffgene.2018.00190&partnerID=40&md5=515f764dc75c043061f8917a81f27e46

80. Stahl BC, Rainey S, Harris E, Fothergill BT. The role of ethics in data governance of large neuro-ICT projects. J Am Med Inform Assoc. 2018 Aug 1;25(8):1099–107.

81. Tosetti P, Hicks RR, Theriault E, Phillips A, Koroshetz W, Draghia-Akli R. Toward an international initiative for traumatic brain injury research. J Neurotrauma. 2013;30(14):1211–22.

82. Brill SB, Moss KO, Prater L. Transformation of the Doctor–Patient Relationship: Big Data, Accountable Care, and Predictive Health Analytics. HEC Forum. 2019;31(4):261–82.

83. Simell BA, Törnwall OM, Hämäläinen I, Wichmann H-E, Anton G, Brennan P, et al. Transnational access to large prospective cohorts in Europe: Current trends and unmet needs. New Biotechnol. 2019;49:98–103.

84. Woolley JP. Trust and Justice in Big Data Analytics: Bringing the Philosophical Literature on Trust to Bear on the Ethics of Consent. Philos Technol. 2019;32(1):111–34.

85. Nicol D, Critchley C, McWhirter R, Whitton T. Understanding public reactions to commercialization of biobanks and use of biobank resources. Soc Sci Med. 2016;162:79–87.

86. Vezyridis P, Timmons S. Understanding the care.data conundrum: New information flows for economic growth. Big Data Soc [Internet]. 2017;4(1). Available from: https://www.scopus.com/inward/record.uri?eid=2-s2.0-85032789524&doi=10.1177%2f2053951716688490&partnerID=40&md5=91ad1cef4d8d6016388a89f2fd47e7a9

87. Friedman MJ, Huber BR, Brady CB, Ursano RJ, Benedek DM, Kowall NW, et al. VA’s National PTSD Brain Bank: a National Resource for Research. Curr Psychiatry Rep [Internet]. 2017;19(10). Available from: https://www.scopus.com/inward/record.uri?eid=2-s2.0-85028338814&doi=10.1007%2fs11920-017-0822-6&partnerID=40&md5=602df8c501cda61bc0cacc22468e7cec

88. Milne R, Brayne C. We need to think about data governance for dementia research in a digital era. Alzheimers Res Ther. 2020 Jan 31;12(1):17.

89. Bollinger JM, Zuk PD, Majumder MA, Versalovic E, Villanueva AG, Hsu RL, et al. What is a Medical Information Commons? J Law Med Ethics. 2019;47(1):41–50.
